# Chiroptical Sensing of Amino Acid Derivatives by Host–Guest Complexation with Cyclo[6]aramide

**DOI:** 10.3390/molecules26134064

**Published:** 2021-07-02

**Authors:** Xuebin Wang, Jiecheng Ji, Zejiang Liu, Yimin Cai, Jialiang Tang, Yunzhi Shi, Cheng Yang, Lihua Yuan

**Affiliations:** Key Laboratory of Radiation Physics and Technology of Ministry of Education, College of Chemistry, Sichuan University, Chengdu 610064, China; 2017222030073@stu.scu.edu.cn (X.W.); jijiecheng_scu@163.com (J.J.); supramolecularsaf@163.com (Z.L.); ymcai20202020@163.com (Y.C.); 2017141231117@stu.scu.edu.cn (J.T.); 2018141231158@stu.scu.edu.cn (Y.S.)

**Keywords:** hydrogen-bonded macrocycle, chiral recognition, amino acid ester, host–guest, circular dichroism

## Abstract

A hydrogen-bonded (H-bonded) amide macrocycle was found to serve as an effective component in the host–guest assembly for a supramolecular chirality transfer process. Circular dichroism (CD) spectroscopy studies showed that the near-planar macrocycle could produce a CD response when combined with three of the twelve L-*α*-amino acid esters (all cryptochiral molecules) tested as possible guests. The host–guest complexation between the macrocycle and cationic guests was explored using NMR, revealing the presence of a strong affinity involving the multi-point recognition of guests. This was further corroborated by density functional theory (DFT) calculations. The present work proposes a new strategy for amplifying the CD signals of cryptochiral molecules by means of H-bonded macrocycle-based host–guest association, and is expected to be useful in designing supramolecular chiroptical sensing materials.

## 1. Introduction

Chirality is ubiquitous in nature, dictating the life processes of creatures on earth, as indicated by, e.g., the function of helicity found in the DNA duplex and peptide assembly [1]. Interestingly, only one kind of chirality is favored in nature. L-amino acid is a typical example of this kind; it constitutes one of the main components of proteins and enzymes [2,3]. Moreover, one homochiral species may demonstrate significantly different biological activity from another. This is particularly important in medicinal chemistry, where a preferential selection of either R- or S-chirality determines the usability of drugs in the clinic [4]. As such, the ability to differentiate and detect molecular chirality is especially important for practical applications, which creates a need for research on more efficient and simple methods for sensing chirality.

Supramolecular optical chirality sensing (SOCS) follows in the wake of the surge of developments in supramolecular chemistry [5]. Features such as its rapid process, low cost and high-throughput, compared to conventional methods of chromatographic analysis, clearly demonstrate the great potential of this strategy. Prominent examples of supramolecular optical systems include bisporphyrins [6,7], coordinating complexes [8], supramolecular assemblies [9,10,11], aromatic boronic acids [12], binaphthyls [13], polymers [14] and interlocked structures [15,16]. The drawback of some of these systems lies in their ineffectiveness at differentiating between chemical structures containing similar functional groups. Another issue hindering the development of SOCS is the lack of spectral responses for some chiral molecules when analyzed using circular dichroism (CD) spectroscopy. These chiral molecules, also named “cryptochiral” molecules, structurally lack a conjugated group adjacent to the stereogenic center [17]. As a result, only weak or negligible Cotton effects have been observed for cryptochiral molecules, making CD spectroscopy insufficiently sensitive to directly analyze such samples. Therefore, there remains a need to search for appropriate supramolecular systems capable of amplifying the CD signals of cryptochiral molecules.

**Scheme 1:**
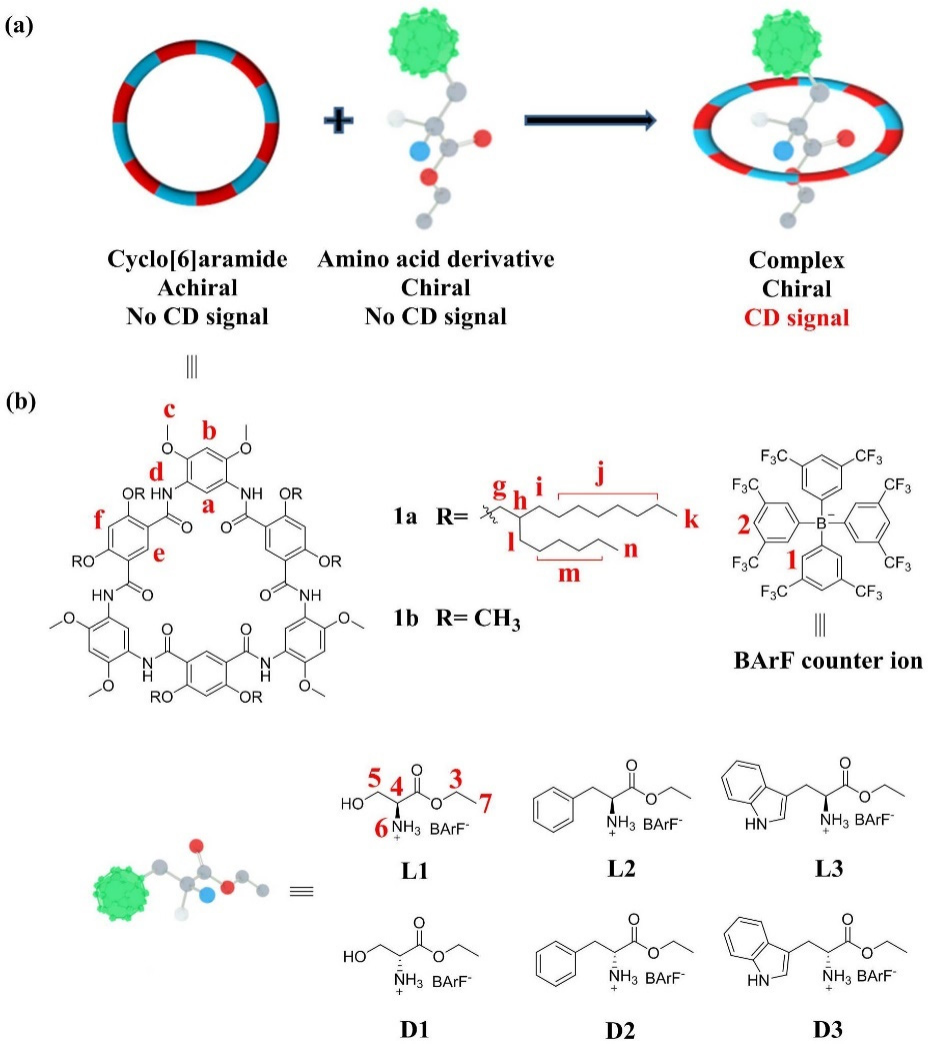
(**a**) Schematic representation of the chiroptical induction of **1a** through complexation with chiral guests. (**b**) Chemical structures of cyclo[6]aramide **1a** and amino acid derivatives **L1**–**L3** and **D1**–**D3**.

The supramolecular strategy provides an alternative to traditional covalent methods of sensing and detecting analytes associated with inherent chiral or achiral properties [18,19,20]. In fact, there are several reports on the use of achiral or dynamically racemic analytes to amplify the chiroptical signal of various cryptochiral molecules, including polymers [21], oligomers [22] and macrocycles [23]. Among these approaches, macrocycle-based host–guest SOCS systems are particularly intriguing [24,25], due to the existence of a large library of macrocycles available for use. However, the difficulty in achieving both guest binding and chiroptical amplification limits its wide application. In 2020, a system based on pillar[5]arene was developed to amplify the CD signals of cryptochiral molecules [26,27,28]. Intriguingly, the planar chirality of pillararenes is induced and stabilized in the presence of a chiral guest by making use of pillararene host–guest complexation, leading to the detection of the cryptochiral molecule by CD spectroscopy. Despite this progress, tapping chiroptical amplifiers that act with a broad substrate scope and undergo highly efficient chirality transfer still remains a formidable task [29]. Inspired by previous research, we hypothesized that cyclo[6]aramide, a type of hydrogen-bonded macrocycle, may function as a candidate host for amplifying the CD signals of cryptochiral molecules, such as *α*-amino acid esters, via the binding of a chiral guest, because the complexation creates an unequal population of enantiomeric conformers through an assembly process, thereby leading to the handedness of the guest and the induced CD signals of the preferred conformers (Scheme 1).

Hydrogen-bonded oligoamide macrocycles [30,31,32] display plenty of interesting H–G behaviors in supramolecular chemistry, as indicated by their uses in recognition [33,34,35], extraction [36], separation [37], transmembrane channels [38], catalysis [39,40] and liquid-crystal materials [41]. Among them, cyclo[6]aramide, a type of hydrogen-bonded amide macrocycle, is particularly noteworthy for the host–guest interactions it demonstrates. This class of macrocycles has a near-planar conformation, containing a cavity decorated with six carbonyl oxygen atoms as binding sites. The electron-rich cavity endows it with a rich H–G chemistry, involving a variety of cationic guest molecules such as dialkylammonium [42], diquat [43], tropylium [44], paraquat [45,46], ferrocenium [47] and pyridinium [48]. We report here that the hydrogen-bonded amide macrocycle host–guest complexation induced chiroptical amplification using cyclo[6]aramide (Scheme 1). The CD responses were found to vary significantly, depending on the side groups of the amino acid guests. The approach proposed in the present study generated a pronounced enantiomeric and structural differentiation of *α*-amino esters when combined with a macrocyclic host. To the best of our knowledge, this represents the first time a hydrogen-bonded macrocycle has been used as a host for sensing the chirality of *α*-amino acid derivatives.

## 2. Results and Discussion

### 2.1. Host–Guest Interaction

Since only those H–G systems which exhibit CD signals of acceptable intensity were considered as potential chiroptical amplification candidates, the interaction between the host and each guest was first examined via screening experiments with the macrocycle **1a** and L-amino acid derivatives **L1**–**L12** (Scheme 1 and Scheme S3), using CD spectroscopy. Results from the screening experiments indicated that, among the 12 L-amino acid derivatives, only **L1** (L-SerD), **L2** (L-PheD) and **L3** (L-TrpD) showed chiroptical amplification features (Figure S13). To follow up on these findings, proton NMR, Job plot and titration experiments were carried out on these three guests only, to collect information on the change of chemical shifts pertinent to H–G interactions, stoichiometry and binding constants.

**Figure 1:**
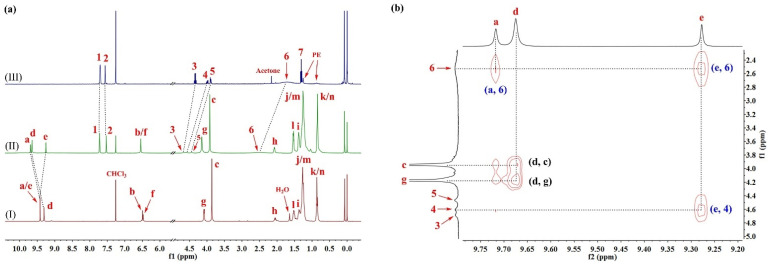
(**a**) Partial ^1^H NMR spectra (400 MHz, CDCl_3_, 298 K) of (I) **1a** (2.0 mM), (II) **1a**⊃**L1** (2.0 mM for each) and (III) **L1** (2.0 mM). (**b**) Expanded 2D NOESY spectrum (600 MHz, CDCl_3_, 298 K, mixing time = 0.4 s) of **1a**⊃**L1** (10 mM for each).

The formation of an H–G complex interaction between cyclo[6]aramide **1a** and **L1** was indicated by a pronounced change in the ^1^H NMR spectra (Figure 1a) upon mixing the macrocyclic host and the guest in chloroform (CDCl_3_). A considerable downfield shift of **L1** protons was observed in such cases. For example, the protons *3*, *4* and *5* of **L1** shifted downfield by 0.33, 0.59 and 0.54 ppm, respectively, after complexation with **1a**. In particular, proton *6* (NH_3_^+^) became so broad that it almost disappeared from the spectrum of the complex, but it was still distinguishable upon scaling the related region between 2.3 and 2.7 ppm. At the same time, proton *2* on the anionic part of **L1** exhibited a slight upfield shift. Commensurate with the changing trend of proton chemical shifts on the guest molecule, the aromatic protons of the host, **1a**, also experienced a change, with both downfield and upfield shifts for interior protons *a*, *d* and *e*. The change of chemical shifts in response to the addition of **L1** indicated site-specific binding by the hydrogen-bonded macrocycle. The binding of **L2** and **L3** in the cavity of **1a** was also observed (Figures S14 and S15). The observation that the macrocycle could bind these guests can be explained by the presence of multiple hydrogen-bonding interactions between the ammonium groups of the amino acid derivatives and the carbonyl oxygen atoms arrayed in the interior cavity of the host [49].

The 2D NOESY technique is a versatile tool used in supramolecular chemistry to pinpoint the binding site of H–G interactions. Thus, NOESY experiments were conducted in CDCl_3_ with the macrocycle **1a** and **L1** (Figure 1b). Cross peaks between the signals, attributable to the interior aromatic protons of **1a** (denoted as *a* and *e*) and the protons of **L1** (denoted as *4* and *6*), were observed. No correlations associated with interactions between the side-chain protons of the host and the internal aromatic protons emerged (Figure S16), suggesting that the complexation most likely occurs when the guest is trapped in the macrocyclic cavity (Figures S17–S20). Results from the Job plot experiments provided a 1:1 stoichiometry for the complex in solution (Figures S21 and S22). The 1:1 binding mode was consistent with the results from the matrix-assisted laser desorption/ionization time-of-flight mass spectrometry (MALDI-TOF-MS) of an equimolar mixture of **1a** and **L1**, where a highest-intensity peak of *m*/*z* = 2470.1285, corresponding to [**1a**⊃**L1**]^+^, was observed, indicating a 1:1 molar ratio for the complex in a gaseous state (Figure S27). **L2** and **L3** followed a similar trend in binding to the host **1a** (Figures S23–S26, S28 and S29).

UV–vis titration experiments were then conducted in CHCl_3_ to further explore the complexation process. The binding affinity of **1a** for **L1** was found to be (5.60 ± 0.01) × 10^2^ M^−1^, using the nonlinear curve-fitting method (Figures S30 and S31). Interestingly, **L2** and **L3** produced binding constants of (3.81 ± 0.03) × 10^3^ M^−1^ and (2.82 ± 0.01) × 10^3^ M^−1^, respectively, in CHCl_3_ (Figures S32–S35), values which were one order of magnitude larger than **L1**’s. Given the structural difference between **L1** and **L2**/**L3** at the β-position of the amino acid derivatives, the higher binding affinity with **L2**/**L3** can be attributed to the π-interaction between aromatic backbones of the host and the aromatic substituent of the guest.

### 2.2. Pseduo-Achiral Host–Chiral Guest Complexation by CD Spectroscopy

**Figure 2:**
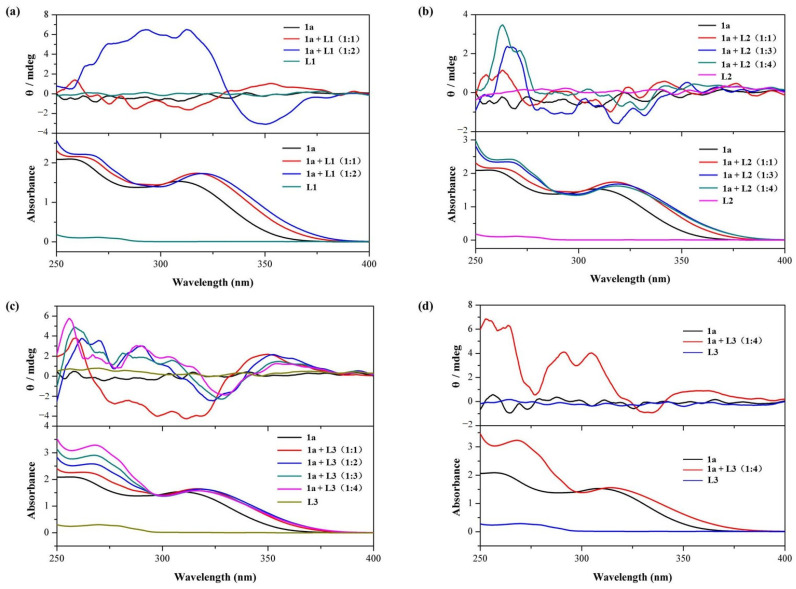
Circular dichroism (CD) spectra and corresponding UV-vis spectra of **1a** (25 μM) in CHCl_3_ at 298 K upon the addition of amino acid derivatives: (**a**) **L1** (0 to 2 equivalents, optical path of cell: 10 mm); (**b**) **L2** (0 to 4 equivalents, optical path of cell: 10 mm); and (**c**) **L3** (0 to 4 equivalents, optical path of cell: 10 mm). (**d**) CD spectra and corresponding UV-vis spectra of **1a** (250 μM) in CHCl_3_ at 298 K upon addition of **L3** (0 and 4 equivalents, optical path of cell: 1 mm).

Having established which three interacting H–G pairs displayed a Cotton effect, the CD spectra of **1a** in the presence of different concentrations of guests **L1**–**L3** were recorded (Figure 2).

Cyclo[6]aramide **1a** alone does not exhibit any CD signals. It is worth noting that **L1** alone also fails to show a CD signal in the wavelength range of 250–400 nm. However, the addition of **L1** (25 μM) to a solution containing **1a** (25 μM) led to the generation of a weak CD signal. Further increasing the concentration of the guest resulted in increased signal intensity, up to a maximum of 6.5 mdeg at 2 equiv. of the guest (Figure 2a). This indicates the occurrence of chiroptical induction of the macrocycle **1a** through its complexation with **L1**, a phenomenon often observed during complexation between an achiral species and a chiral molecule [50]. In supramolecular chemistry, host–guest interactions play a crucial role in determining how chirality is transferred from guest to host [51]. As discussed in Section 2.1, the electron-rich cavity is prone to capturing a cationic component, such as the ammonium part of **L1**.

Similar results were obtained when **L2** was added to the host **1a** (Figure 2b). A CD signal for **1a** appeared, with a maximum of 3.5 mdeg at 263 nm (Table 1). In the case of **L3**, the CD signal exhibited a positive, but relatively marked, Cotton effect, with signal intensity reaching around 6 mdeg. This strong positive Cotton effect could be attributed to the alteration of **1a**’s conformation, induced more pronouncedly by **L3** (Figure 2c) compared to **L2**. The bulkier residue (rigid indole aromatic ring) of **L3** is likely to be responsible for this result. However, when **L3** (1000 μM) was added to a solution containing **1a** (250 μM), there was no significant enhancement of the induced CD signal intensity (Figure 2d). This indicates that increasing the concentrations of **1a** and **L3** does not significantly enhance the intensity of the CD signal induced by chiroptical sensing.

In addition, the CD spectra of mixtures containing **1a** and the corresponding D-enantiomers of the amino acid esters of serine **D1** (D-SerD), phenylalanine **D2** (D-PheD) and tryptophan **D3** (D-TrpD) were recorded (Figures S36–S38, respectively). The CD spectral profiles of **1a**⊃**L1**/**D1** were found to be symmetrical, with a host–guest stoichiometric ratio of 1:2, and similarly, the spectra of **1a**⊃**L2**/**D2** (1:2) and **1a**⊃**L3**/**D3** (1:4) were also symmetrical.

**Figure 3:**
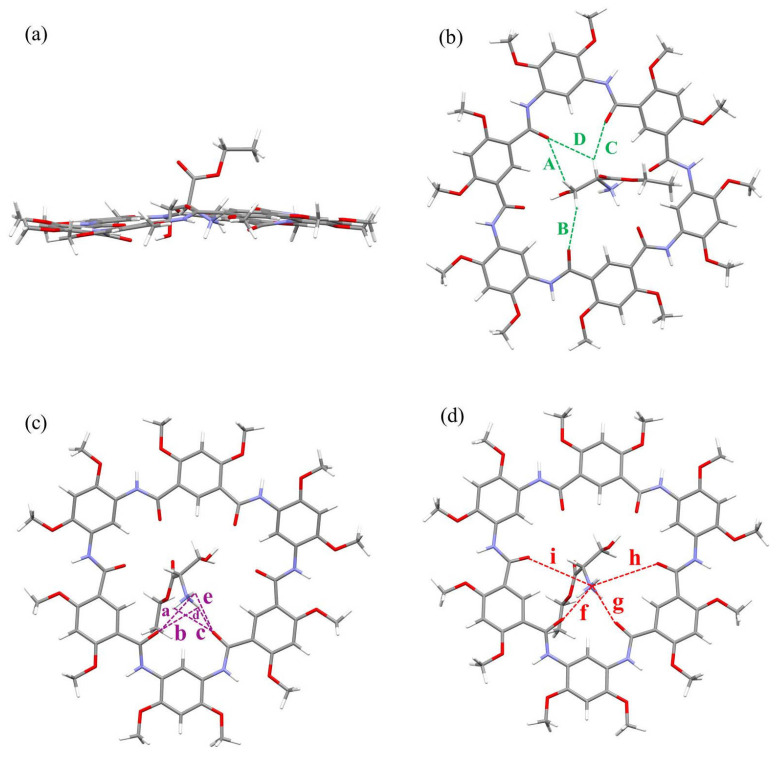
Side view (**a**) and top views (**b**–**d**) of optimized geometry of complex **1b**⊃**L1** at the B3LYP/6-311G (d, p) level (gray = C, white = H, red = O and blue = N). All side chains have been replaced by methyl groups for simplicity, and the counterion tetrakis (3,5-bis(trifluoromethyl)phenyl) borate anion (BArF^−^) is omitted for clarity. The green dashed lines indicate C-H∙∙∙O hydrogen bonds A–D, where A = 2.851 Å (121.95°), B = 2.628 Å (140.95°), C = 2.446 Å (145.52°), D = 3.108 Å (123.54°). The purple dashed lines indicate N-H∙∙∙O hydrogen bonds a–e, where a = 3.274 Å (52.99°), b = 3.058 Å (64.77°), c = 3.047 Å (68.12°), d = 1.862 Å (154.20°), e = 3.241 Å (57.73°). The red dashed lines indicate N^+^∙∙∙O cation–dipole interactions f–i, where f = 2.779 Å, g = 2.829 Å, h = 4.554 Å and i = 4.151 Å.

Inspection of the chemical structure of **L1**–**L3** suggests that the presence of hydroxyl groups and aromatic moieties attached to the β-position seems to be important for chiroptical induction, since all other chiral *α*-ammonium ester BArF salts failed to show any CD signal or only exhibited signals of negligible intensity. To collect more information about the transfer of chirality from guest to host, computational simulations based on the DFT method were performed at the B3LYP/6-311G (d, p) level. The results indicated that the complexes **1b**⊃**L1**–**L3** were built by assembling the macrocyclic molecule of near-planar conformation and the amino acid motif in a threaded orthogonal binding arrangement. Multiple C-H∙∙∙O H-bonds, N-H∙∙∙O H-bonds and N^+^∙∙∙O ion–dipole interactions were present in the modelling structures that assisted the H–G complex formation (Figure 3, Figures S39 and S40). For example, in the case of **L1**, there were four C-H∙∙∙O hydrogen bonds, five N-H∙∙∙O hydrogen bonds and four N^+^∙∙∙O cation-dipole interactions in the complex (Figure 3). With this in mind, we speculated that the host **1a** may undergo a slight conformational change upon binding to the guest, resulting in variation in the dihedral angles between the planes of adjacent phenyl groups. The variation in the dihedral angles of molecular constituents is one of the key parameters used to indicate stimulus-induced chirality transfer in H–G complexes [52]. However, a convincing possible pathway for the production of chirality transfer will be studied in future work.

## 3. Materials and Methods

### 3.1. Materials and Reagents

Ethyl L-tryptophanate hydrochloride was purchased from Tokyo Chemical Industry Co. Ltd. (Tokyo, Japan). L-arginine ethyl ester dihydrochloride, L-cysteine ethyl ester hydrochloride and CDCl_3_ were purchased from Energy Chemical (Shanghai, China). Ethyl L-phenylalaninate hydrochloride, ethyl L-serinate hydrochloride, ethyl L-alaninate hydrochloride, ethyl L-valinate hydrochloride, ethyl L-leucinate hydrochloride, L-isoleucine ethyl ester hydrochloride, ethyl L-methionate hydrochloride, diethyl L-glutamate hydrochloride, diethyl L-aminosuccinate hydrochloride and sodium tetrakis (3,5-bis(trifluoromethyl)phenyl) borate were purchased from Adamas Reagent, Ltd. (Shanghai, China). Ethyl D-serinate hydrochloride, ethyl D-phenylalaninate hydrochloride and ethyl D-tryptophanate hydrochloride were purchased from Bide Pharmatech Ltd. (Shanghai, China). HPLC chloroform was purchased from Chron Chemicals (Chengdu, China). All reagents purchased from commercial suppliers were used without further purification.

### 3.2. Experimental Methods

The ^1^H NMR and ^13^C{^1^H} NMR spectra were recorded on an AVANCE III HD nuclear magnetic resonance spectrometer (Bruker, Karlsruhe, Germany) in CDCl_3_ at 298 K (400 MHz for ^1^H NMR and 101 MHz for ^13^C{^1^H} NMR). The 2D NOESY spectra were acquired using an AV II-600 MHz nuclear magnetic resonance spectrometer in CDCl_3_ at 298 K (Bruker, Karlsruhe, Germany). The ESI-HRMS spectra of **L3**, **D1**, **D2** and **D3** were acquired using a LCMS-IT-TOF mass spectrometer (SHIMADZU, Kyoto, Japan). Host–guest stoichiometries were recorded on an AXIMA Performance MALDI-TOF Mass Spectrometer (SHIMADZU, Kyoto, Japan). Stoichiometries based on Job plot experiments and binding constants with UV–vis titration were obtained using a UV-2450 UV–visible spectrophotometer (SHIMADZU, Kyoto, Japan). The binding constants were obtained by nonlinear fitting with a method named Nelder–Mead on the website supramolecular.org (accessed on 16th February 2021). Herein, the changes in UV–vis spectra at 370 nm were plotted along with the added guest equivalent. Then, the obtained plots were fitted with the nonlinear curve method by selecting the option “UV 1:1”.

The CD and corresponding UV–vis spectra were recorded on a Jasco model J-1500 spectrometer (JASCO Corporation, Tokyo, Japan) with a scan speed of 200 nm/min, a data pitch of 0.1 nm, a bandwidth of 2 nm and optical path length cells of 10 mm and 1 mm. All spectra were averaged over four measurements. Taking **1a**⊃**L1** as an example, the CD and UV–vis spectra of 2 mL of 25 µM **1a** in CHCl_3_ were measured with the sequential addition of a 10 mM **L1** solution. The guest was added to a CHCl_3_ solution of **1a** (25 μM), in order to keep the concentration of the host **1a** solution almost constant.

### 3.3. Synthesis of Cyclo[6]aramide and Amino Acid Derivatives

Cyclo[6]aramide **1a** was synthesized according to previous references [53,54].

All the amino acid derivatives were prepared as previously reported [26], according to the reactions described in Schemes S1 and S2, among which **L3**, **D1**, **D2** and **D3** were newly synthesized (Figures S1–S12). A typical procedure, as represented by the synthesis of guests **L3**, **D1**, **D2** and **D3,** was undertaken, as follows.

A mixture of amino acid ester hydrochloride (1.0 equiv., 100 mg) and sodium tetrakis [3,5-bis(trifluoromethyl)phenyl] borate (NaBArF) (1.1 equiv.) was added to chloroform (40 mL) in a 100 mL round-bottom flask. This was then stirred at 35 ℃ for 24 h. The progress of the reaction was monitored by taking an aliquot from the clear solution. The anion exchange reaction was deemed complete when no further precipitation (AgCl) was observed upon addition of an aqueous solution of AgNO_3_ to the aliquot. The resulting white precipitate was removed by filtering twice. The solvent of the filtrate was subjected to rotary evaporation under reduced pressure. The resultant yellow viscous solid was dried in a vacuum at 45 ℃ for two days, to generate the target products.

**Compound L3** (241 mg, 59%): ^1^H NMR (400 MHz, CDCl_3_, 298 K) δ 8.23 (s, 1H), 7.70 (s, 8H), 7.53 (s, 4H), 7.45–7.42 (m, 2H), 7.34–7.30 (m, 1H), 7.23–7.19 (m, 1H), 7.02–7.01 (d, *J* = 2.5 Hz, 1H), 4.40–4.34 (q, *J* = 7.2 Hz, 2H), 4.24–4.21 (dd, *J* = 9.3, 4.7 Hz, 1H), 3.65–3.60 (dd, *J* = 15.6, 4.6 Hz, 1H), 3.26–3.20 (dd, *J* = 15.6, 9.3 Hz, 1H), 1.40–1.36 (t, *J* = 7.2 Hz, 3H); ^13^C{^1^H} NMR (101 MHz, CDCl_3_, 298 K) δ 167.87, 136.57, 134.78, 129.09, 128.78, 128.59, 125.88, 125.80, 123.98, 123.94, 123.17, 121.15, 117.56, 117.21, 112.29, 105.18, 64.55, 54.25, 26.10, 13.86. ESI-HRMS: *m*/*z* calcd. for C_45_H_29_BF_24_N_2_O_2_ [M-BArF^−^]^+^, 233.1285, found: 233.1282.

**Compound D1** (382 mg, 65%): ^1^H NMR (400 MHz, CDCl_3_, 298 K) δ 7.69 (s, 8H), 7.55 (s, 4H), 4.30–4.25 (q, *J* = 7.1 Hz, 2H), 4.03–3.99 (dd, *J* = 12.2, 6.3 Hz, 1H), 3.97–3.93 (m, 2H), 1.26–1.23 (t, *J* = 7.1 Hz, 3H); ^13^C{^1^H} NMR (101 MHz, CDCl_3_, 298 K) δ 166.22, 162.38, 161.88, 161.39, 160.89, 134.76, 129.41, 129.09, 128.78, 128.59, 128.50, 125.88, 123.17, 120.47, 117.60, 64.81, 58.94, 55.10, 13.57. ESI-HRMS: *m*/*z* calcd. for C_37_H_24_BF_24_NO_3_ [M-BArF^−^]^+^, 134.0812, found: 134.0812.

**Compound D2** (285 mg, 62%): ^1^H NMR (400 MHz, CDCl_3_, 298 K) δ 7.70 (s, 8H), 7.54 (s, 4H), 7.36–7.32 (dd, *J* = 4.6, 1.9 Hz, 3H), 7.12–7.09 (dd, *J* = 6.7, 2.9 Hz, 2H), 4.34–4.27 (qd, *J* = 7.2, 2.4 Hz, 2H), 4.12–4.09 (dd, *J* = 9.5, 4.5 Hz, 1H), 3.45–3.41 (dd, *J* = 14.9, 4.5 Hz, 1H), 3.06–3.00 (dd, *J* = 14.9, 9.5 Hz, 1H), 1.31–1.27 (t, *J* = 7.1 Hz, 3H); ^13^C{^1^H} NMR (101 MHz, CDCl_3_, 298 K) δ 167.77, 162.40, 161.90, 161.41, 160.91, 134.79, 131.64, 130.14, 129.33, 129.10, 129.07, 129.04, 128.86, 128.78, 128.76, 128.73, 128.44, 125.91, 123.20, 120.50, 117.61, 117.57, 117.53, 64.60, 55.32, 35.95, 13.64. ESI-HRMS: *m*/*z* calcd. for C_43_H_28_BF_24_NO_2_ [M-BArF^−^]^+^, 194.1176, found: 194.1176.

**Compound D3** (228 mg, 56%): ^1^H NMR (400 MHz, CDCl_3_, 298 K) δ 8.18 (s, 1H), 7.72 (s, 8H), 7.53 (s, 4H), 7.45–7.43 (d, *J* = 8.0 Hz, 1H), 7.38–7.36 (d, *J* = 8.2 Hz, 1H), 7.28–7.24 (m, 1H), 7.19–7.15 (m, 1H), 7.01–7.00 (d, *J* = 2.5 Hz, 1H), 4.35–4.30 (q, *J* = 7.1 Hz, 3H), 4.26–4.22 (dd, *J* = 9.4, 4.6 Hz, 1H), 3.65–3.60 (dd, *J* = 15.5, 4.6 Hz, 1H), 3.25–3.19 (dd, *J* = 15.6, 9.4 Hz, 1H), 1.33–1.30 (t, *J* = 7.1 Hz, 3H); ^13^C{^1^H} NMR (101 MHz, CDCl_3_, 298 K) δ 168.09, 162.42, 161.92, 161.43, 160.93, 136.56, 134.81, 129.38, 129.08, 129.05, 128.77, 128.74, 128.64, 128.45, 125.93, 125.60, 124.07, 123.97, 123.23, 121.27, 120.52, 117.55, 117.04, 112.40, 105.26, 64.70, 54.45, 26.33, 13.69. ESI-HRMS: *m*/*z* calcd. for C_45_H_29_BF_24_N_2_O_2_ [M-BArF^−^]^+^, 233.1285, found: 233.1285.

### 3.4. DFT Calculations

DFT calculations for the geometrical optimizations were carried out using the Gaussian 09 program [55]. To reduce the high cost of calculation, due to the large number of atoms in the host–guest complex, all substituents (R) on the periphery of cyclo[6]aramide were replaced by methyl groups, producing compound **1b**, which was used for computational purposes.

## 4. Conclusions

In summary, the chiroptical amplification of amino acid derivatives can be achieved by using a hydrogen-bonded amide macrocycle. In particular, three of the chiral amino acid esters were found to exhibit chiroptical induction upon host–guest complexation in chloroform, as indicated by CD spectroscopy. Only those guest molecules containing different π-aromatic systems (phenyl and indolyl) or the polar group (OH) were found to produce a Cotton effect. The results of the DFT calculations suggest that the chiroptical amplification could be attributed to the assembly associated with the change of dihedral angles of the phenyl units in the macrocyclic backbone, which occur as a result of the H–G interaction. This work not only provides a novel supramolecular chirality transfer system induced by local conformational change of the macrocycle due to the multi-point recognition of guests, but also paves the way for designing other H-bonded macrocycle-based chiral transfer components for creating supramolecular chiroptical sensing materials.

## Figures and Tables

**Table 1:** Results of UV–vis spectroscopic titration for binding constant determination and CD spectroscopy of **1a** with **L1**–**L3** in HPLC chloroform at 298 K.

Complex	Stoichiometry ^a^	*Ka* (M^−1^) ^b^	R^2^	CD λ_max_/λ_min_ (nm) ^c^
**1a**⊃**L1**	1:1	(5.60 ± 0.01) × 10^2^	0.9968	312/350
**1a**⊃**L2**	1:1	(3.81 ± 0.03) × 10^3^	0.9902	263/330
**1a**⊃**L3**	1:1	(2.82 ± 0.01) × 10^3^	0.9987	256/330

^a^ The 1:1 host–guest stoichiometry of each complex was measured by Job plot experiment. ^b^ Binding constant values *Ka* of each complex. ^c^ Maximum and minimum of CD spectra with the highest equivalence of guests.

## Data Availability

Not applicable.

## Supplementary Materials

The following are available online. Schemes S1 and S2: synthetic route for compounds **L3**, **D1**, **D2** and **D3**; Figures S1–S12: ^1^H NMR, ^13^C{^1^H} NMR and ESI-HRMS spectra of compounds **L3**, **D1**, **D2** and **D3** in CDCl_3_ at 298 K; Scheme S3: chemical structures of compounds **L4**–**L****12**; Figure S13: stacked CD spectra of **1a** and **L1–****L12** in the presence of **1a**; Figures S14 and S15: stacked ^1^H NMR spectra of **1a**⊃**L2** and **1a**⊃**L3**; Figures S16–S20: 2D NOESY spectra; Figures S21–S26: stacked UV–vis spectra of Job plot experiments and corresponding curves of host–guest stoichiometries between **1a** and **L1**–**L****3,** respectively; Figures S27–S29: MALDI-TOF-MS of **1a** and **L1**–**L****3,** respectively; Figures S30–S35: UV–vis titration and corresponding fitting curves of **1a** and **L1**–**L****3**; Figures S36–S38: symmetrical CD spectral profiles of **1a**, **1a**⊃L/D-amino acid esters and L/D-amino acid esters; Figures S39 and S40: DFT simulations of **1****b**⊃**L2** and **1b**⊃**L3**.

## Sample Availability

Samples of the compounds **1a**, **L1**–**L3** and **D1**–**D3** are available from the authors.
