# Supplementary material for: Chiroptical Sensing of Amino Acid Derivatives by Host–Guest Complexation with Cyclo[6]aramide
Source: Molecules. 2021 Jul 2;26(13):4064. doi: 10.3390/molecules26134064 (PMC8271771; doi:10.3390/molecules26134064)
Supplement: Supplementary file 1 [file molecules-26-04064-s001.zip › molecules-1247034-supplementary.pdf]

Supporting Information

Chiroptical Sensing of Amino Acid Derivatives by Host-Guest Complexation with Cyclo[6]aramide

Xuebin Wang, Jiecheng Ji, Zejiang Liu, Yimin Cai, Jialiang Tang, Yunzhi Shi, Cheng Yang* and Lihua Yuan*

College of Chemistry, Key Laboratory of Radiation Physics and Technology of Ministry of Education, Sichuan University, Chengdu 610064, China; 2017222030073@stu.scu.edu.cn (X.W.); jijiecheng_scu@163.com (J.J.); supramolecularsaf@163.com (Z.L.); ymcai20202020@163.com (Y.C.); 2017141231117@stu.scu.edu.cn (J.T.); 2018141231158@stu.scu.edu.cn (Y.S.)

* *Correspondence: yangchengyc@scu.edu.cn (C.Y.); lhyuan@scu.edu.cn (L.Y.)*

Table of Contents

1. Synthetic route of novel compounds........1

2. ^{1}H NMR and ^{13}C{^{1}H} NMR spectra of novel compounds........2

3. ESI-HRMS spectrum of novel compounds........7

4. Chemical structures of **L4-L12**........8

5. Stacked CD spectra between **1a** and **L1-L12**........9

6. Stacked ^{1}H NMR spectra of **1a⊃L2-L3**........10

7. 2D NOESY spectra........11

8. Binding ratios determination between **1a** and **L1-L3**........15

   8.1 Job Plot experiments........15

   8.2 MALDI-TOF-MS experiments........18

9. Binding constants determination between **1a** and **L1-L3**........19

10. Symmetrical CD spectral profiles of **1a**, **1a**⊃L/D-amino acid esters and L/D-amino acid esters........22

11. DFT simulations........24

1. Synthetic route of novel compounds

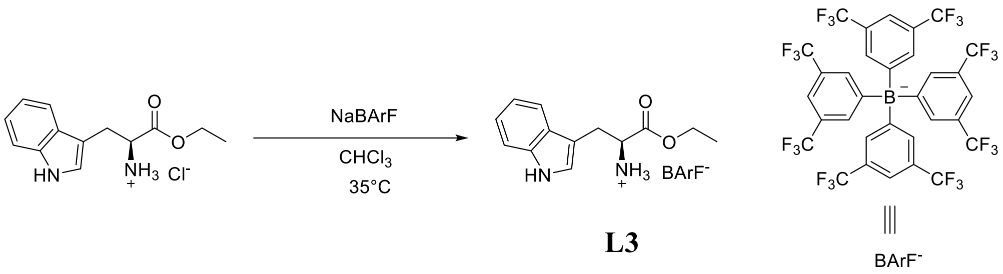

Scheme S1. Synthetic route for compound **L3**.

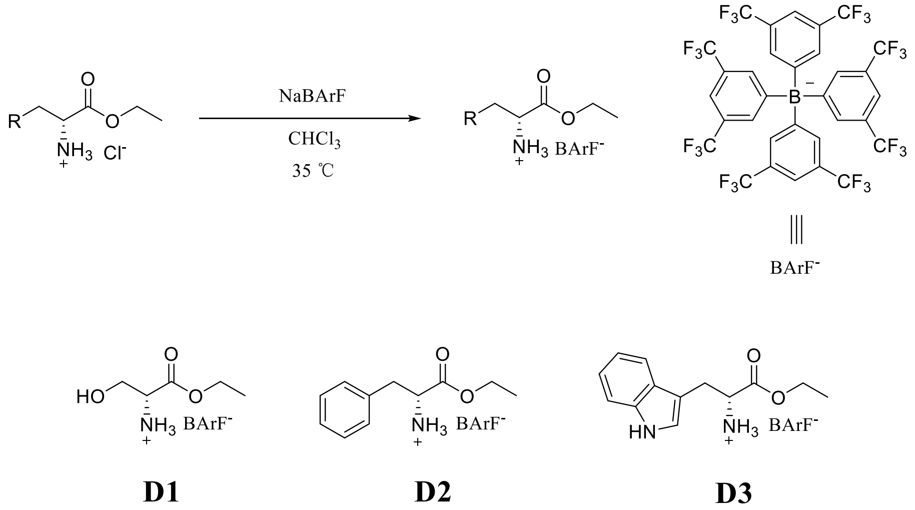

Scheme S2. Synthetic route for compounds **D1**, **D2** and **D3**.

2. ^{1}H NMR and ^{13}C{^{1}H} NMR spectra of novel compounds

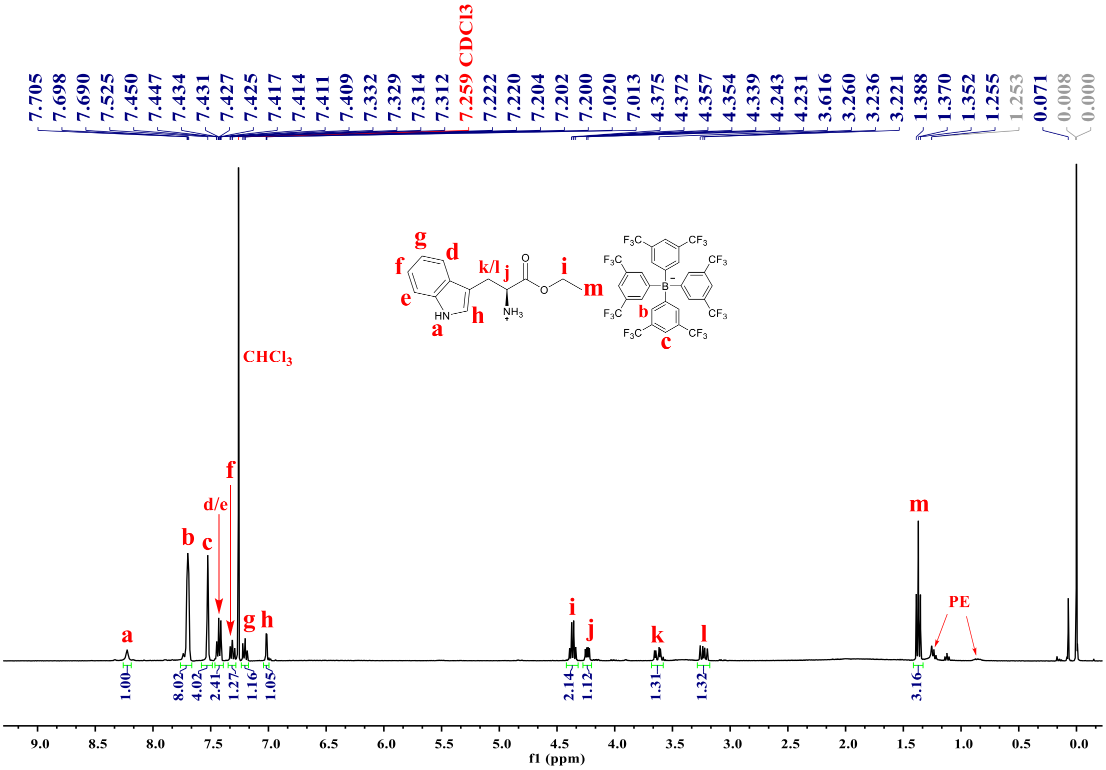

Figure S1. ^{1}H NMR spectrum of compound **L3** (400 MHz, $CDCl_3$, 298 K).

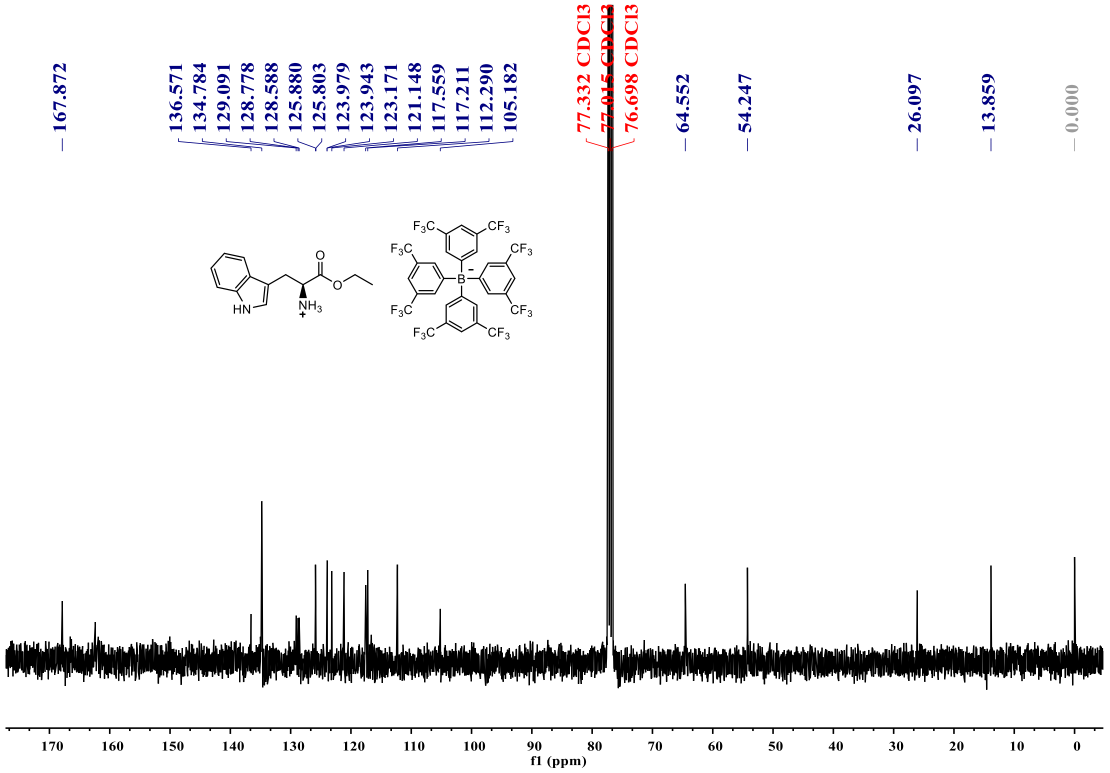

Figure S2. $^{13}C\{^1H\}$ NMR spectrum of compound **L3** (101 MHz, $CDCl_3$, 298 K).

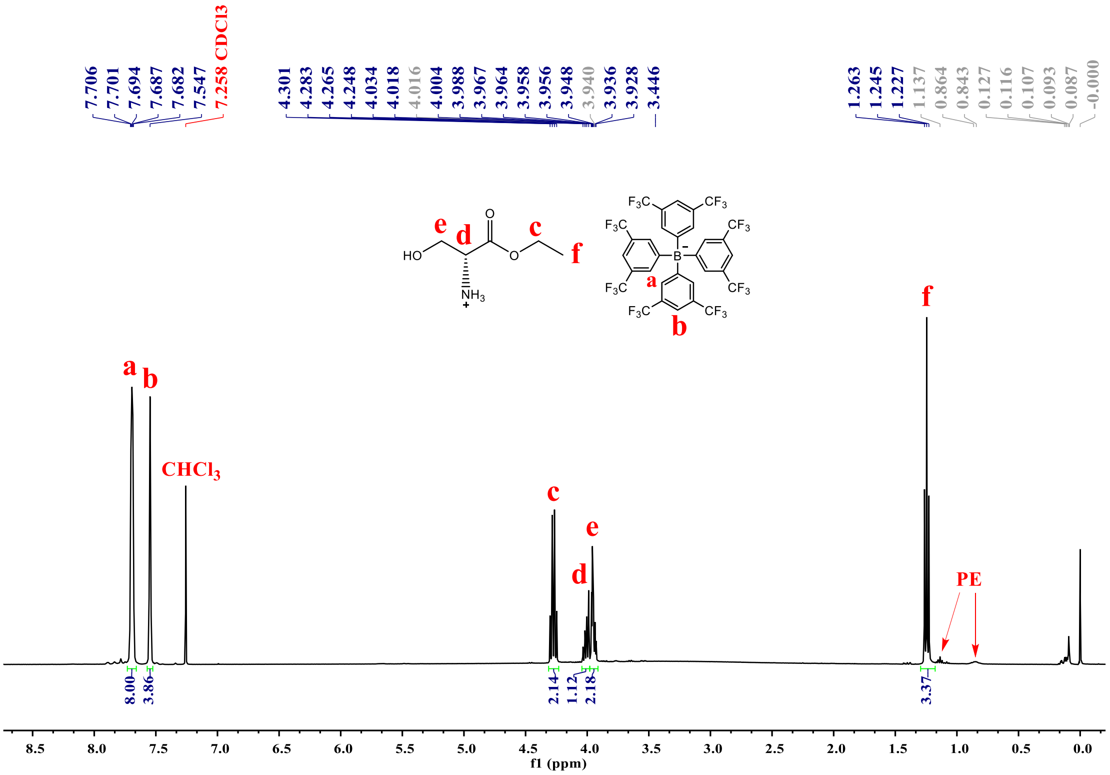

Figure S3. ^1H NMR spectrum of compound **D1** (400 MHz, $CDCl_3$, 298 K).

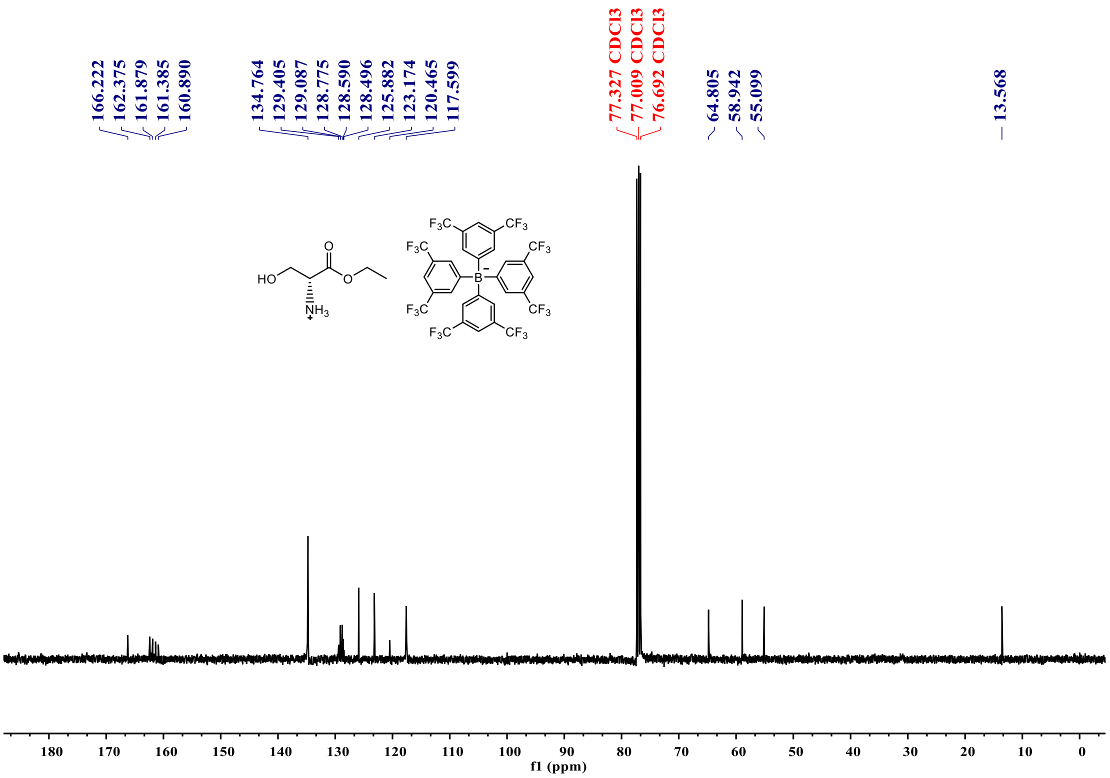

Figure S4. $^{13}C\{^{1}H\}$ NMR spectrum of compound **D1** (101 MHz, $CDCl_3$, 298 K).

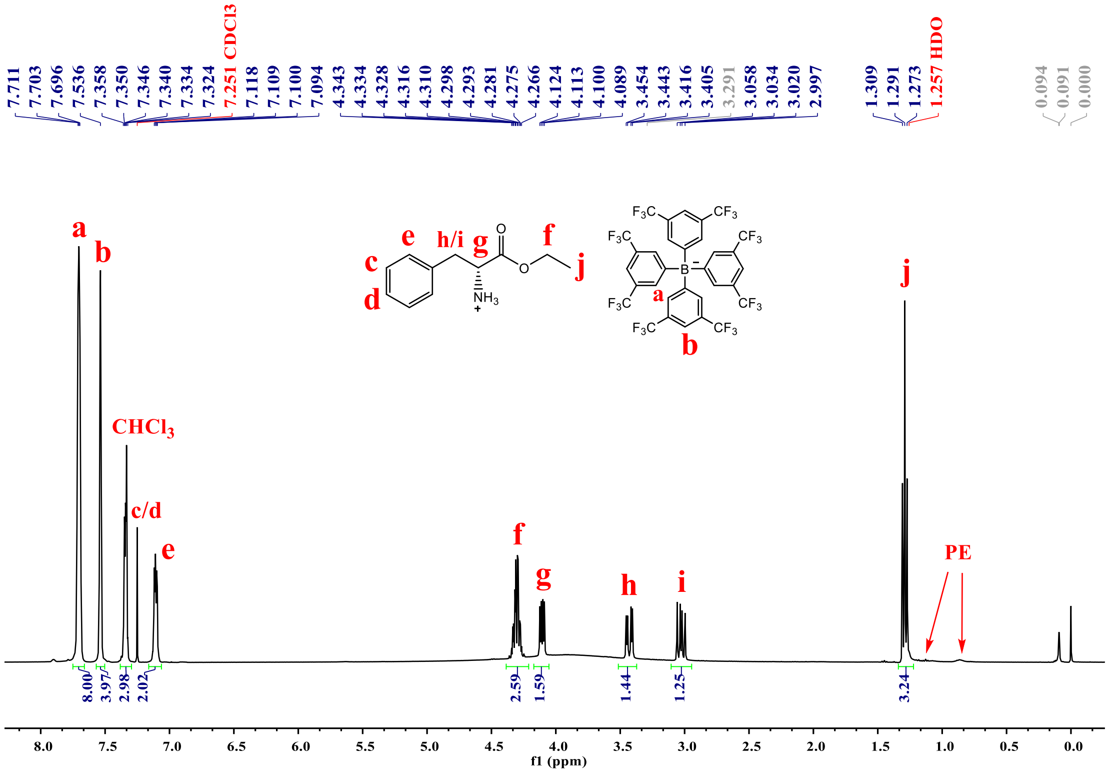

Figure S5. ^{1}H NMR spectrum of compound **D2** (400 MHz, $CDCl_3$, 298 K).

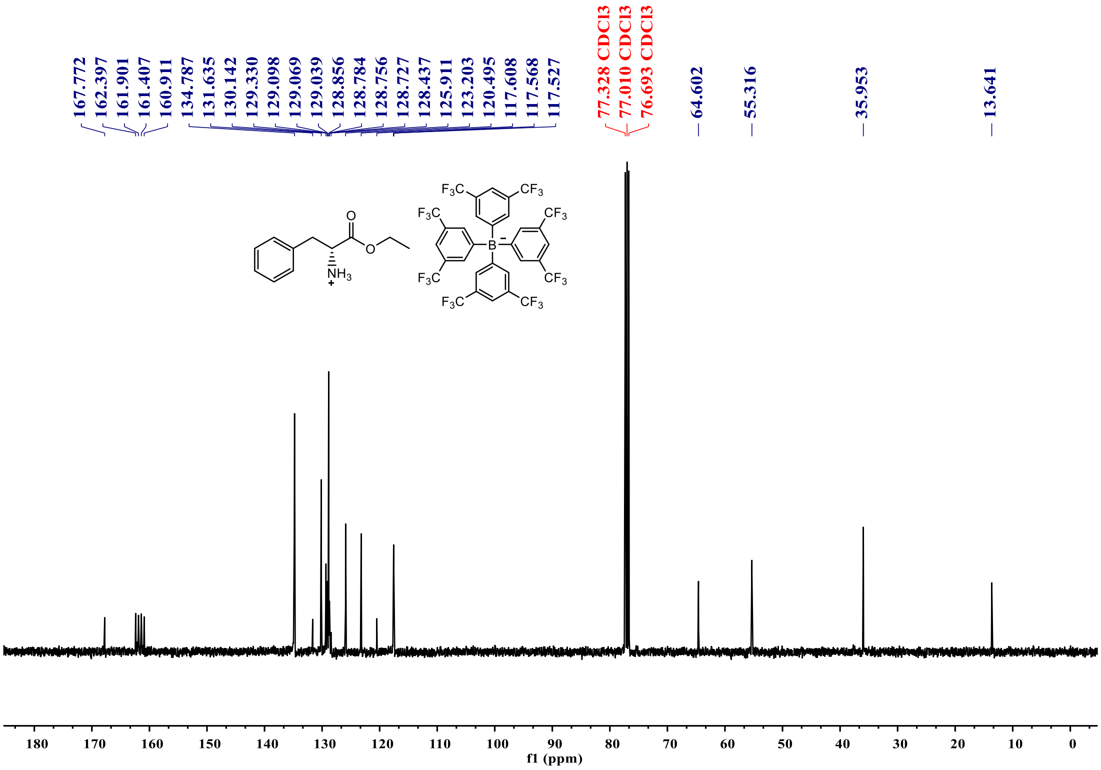

Figure S6. $^{13}C\{^{1}H\}$ NMR spectrum of compound **D2** (101 MHz, $CDCl_3$, 298 K).

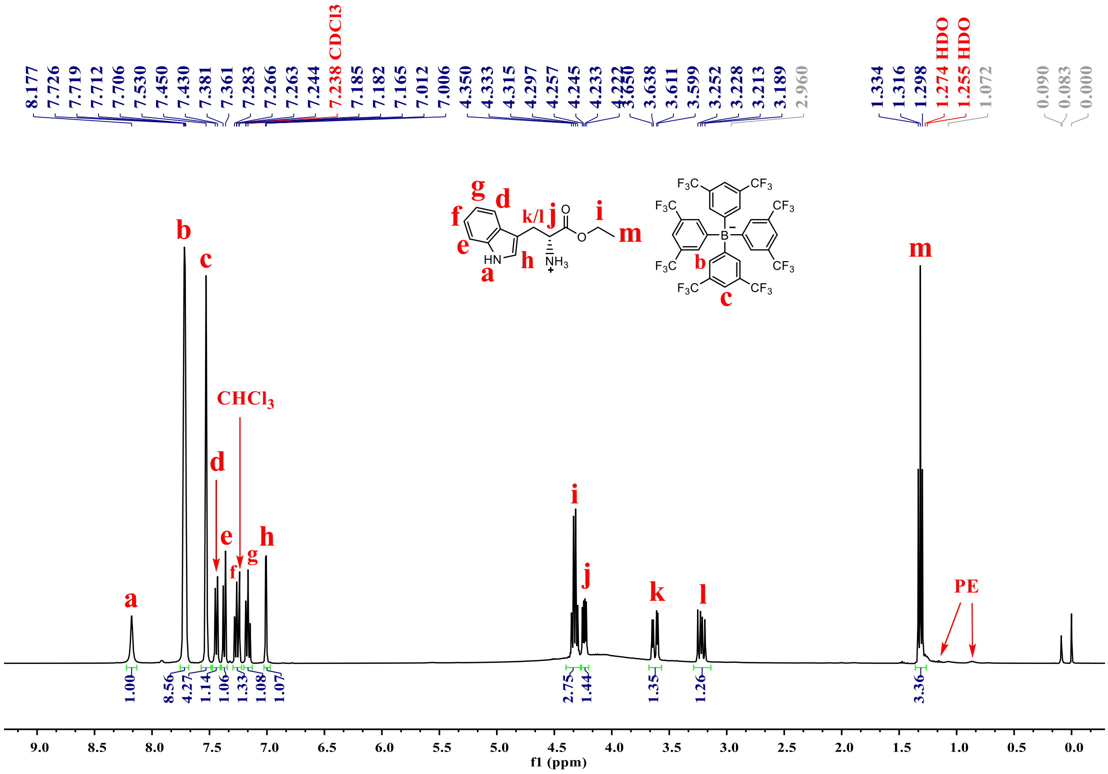

Figure S7. ^{1}H NMR spectrum of compound **D3** (400 MHz, $CDCl_3$, 298 K).

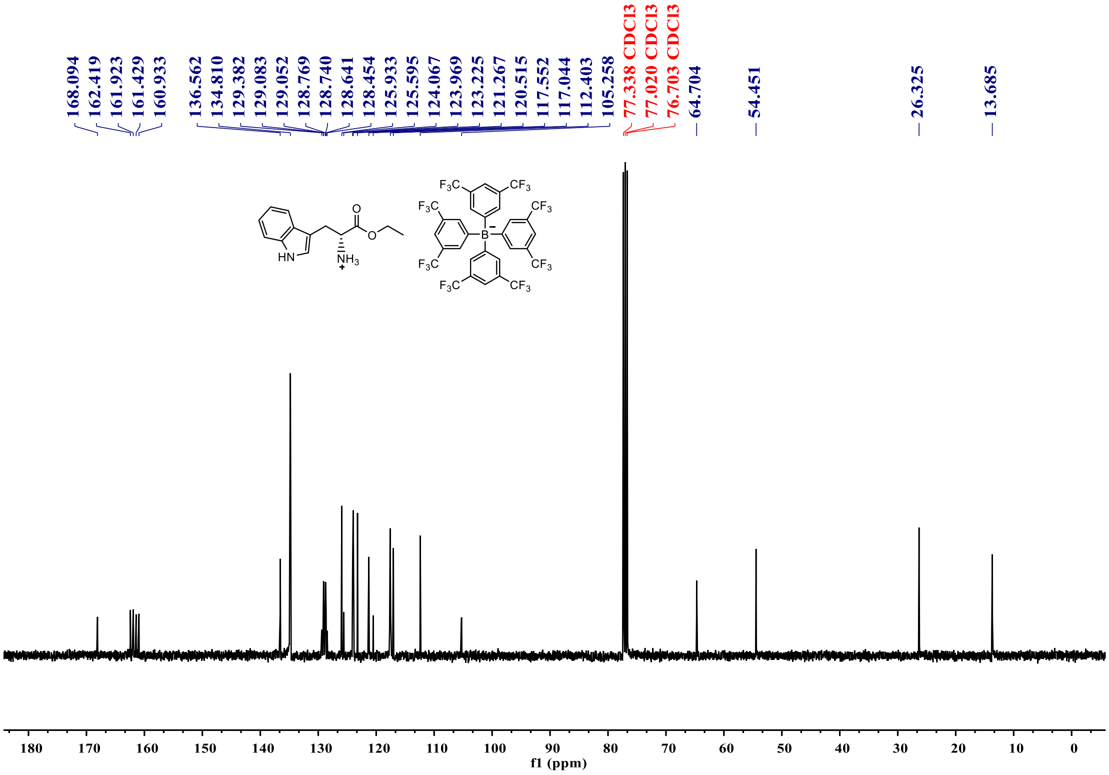

Figure S8. ^{13}C{^{1}H} NMR spectrum of compound **D3** (101 MHz, $CDCl_3$, 298 K).

3. ESI-HRMS spectrum of novel compounds

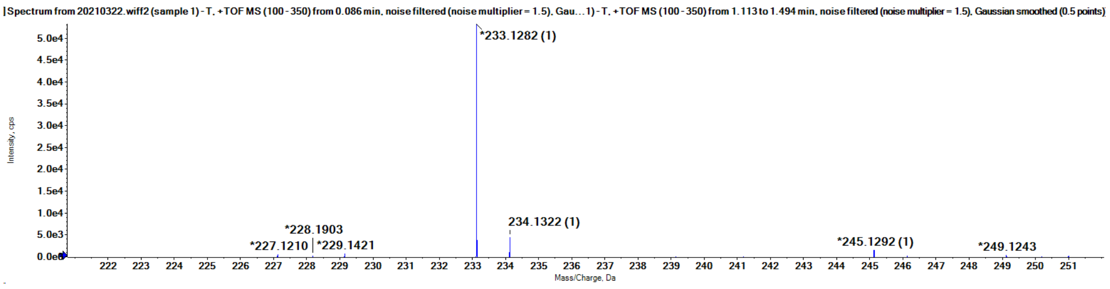

Figure S9. ESI-HRMS spectrum of compound **L3**.

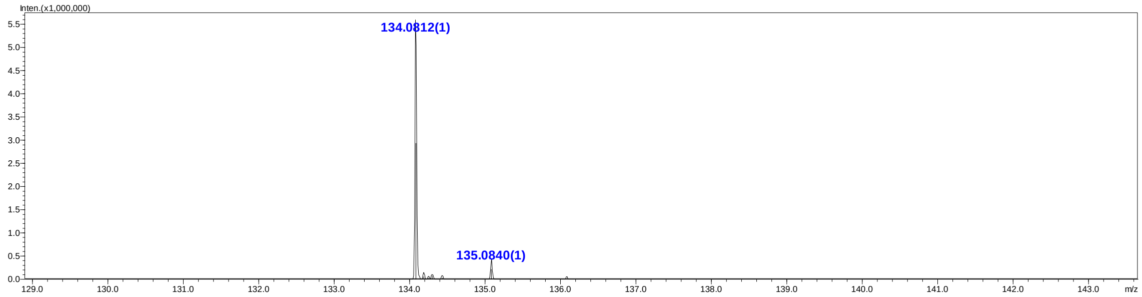

Figure S10. ESI-HRMS spectrum of compound **D1**.

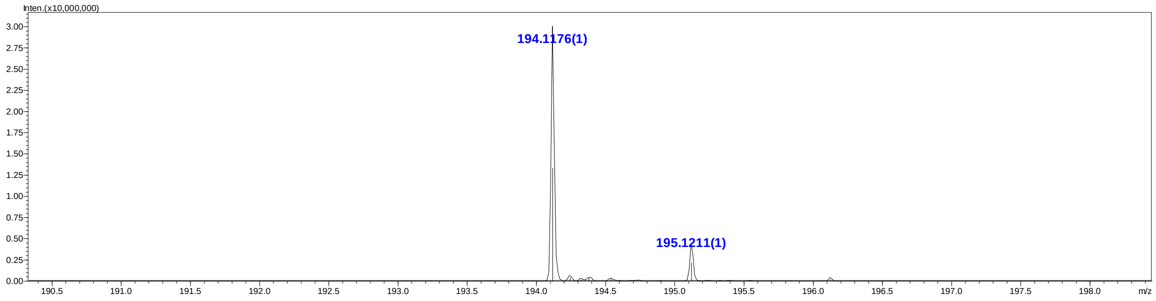

Figure S11. ESI-HRMS spectrum of compound **D2**.

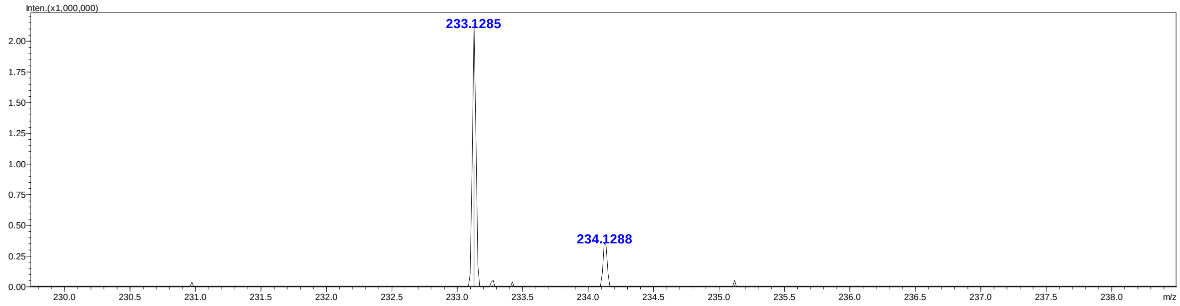

Figure S12. ESI-HRMS spectrum of compound **D3**.

4. Chemical structures of L4-L12

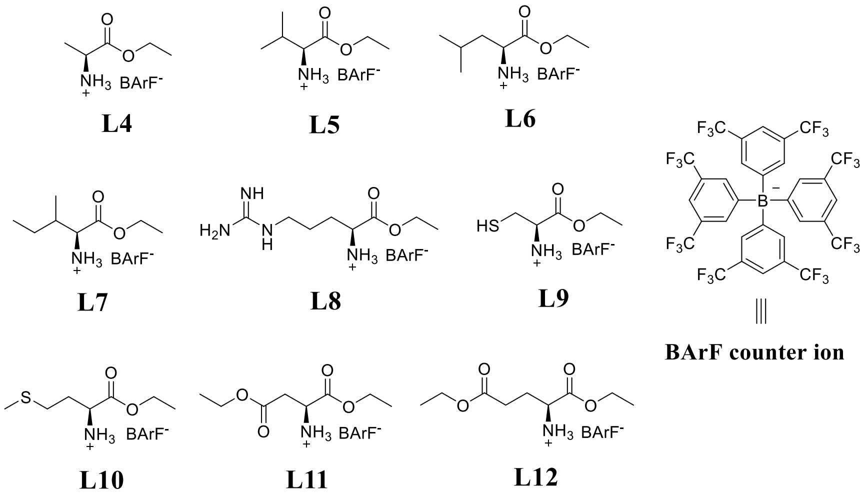

Scheme S3. Chemical structures of compounds **L4-L12**.

5. Stacked CD spectra between 1a and L1-L12

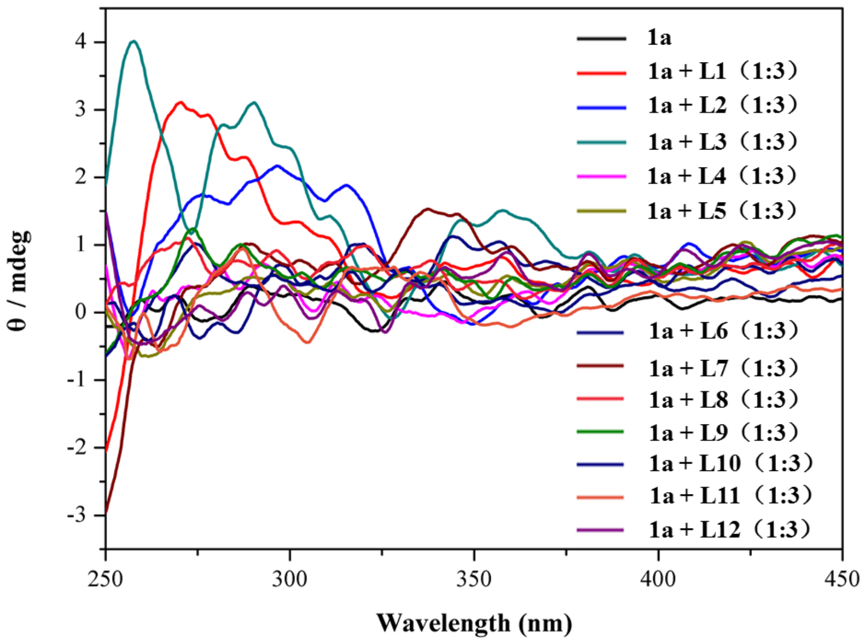

Figure S13. Stacked CD spectra of cyclo[6]aramide **1a** (25 μM) and chiral guests **L1-L12** (74 μM) in the presence of host **1a** (25 μM) measured in HPLC chloroform at 298 K respectively (optical path of cell: 10 mm).

6. Stacked ^{1}H NMR spectra of 1a⊃L2-L3

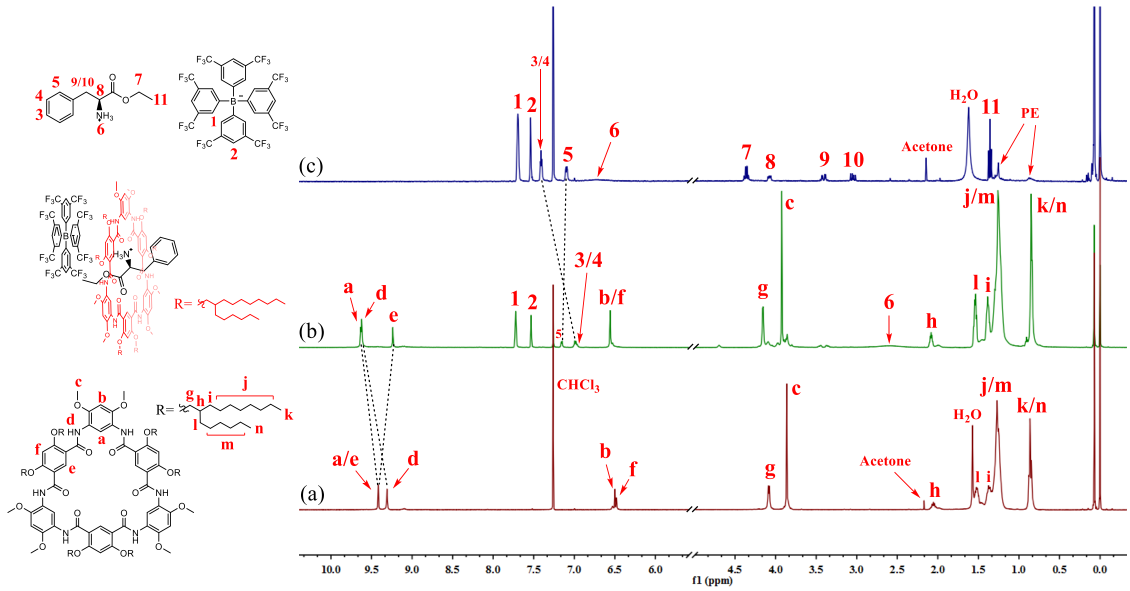

Figure S14. Stacked ^{1}H NMR spectra (400 MHz, $CDCl_3$, 298 K) of (a) 2 mM **1a**, (b) 2 mM **1a** and 2 mM **L2**, (c) 2 mM **L2**.

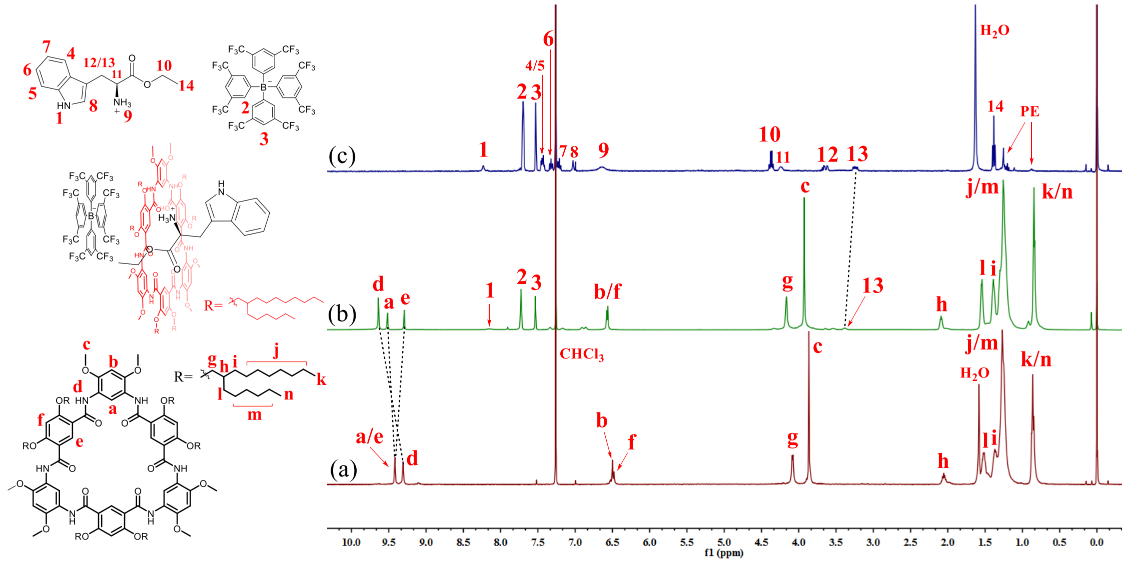

Figure S15. Stacked ^{1}H NMR spectra (400 MHz, $CDCl_3$, 298 K) of (a) 2 mM **1a**, (b) 2 mM **1a** and 2 mM **L3**, (c) 2 mM **L3**.

7. 2D NOESY spectra

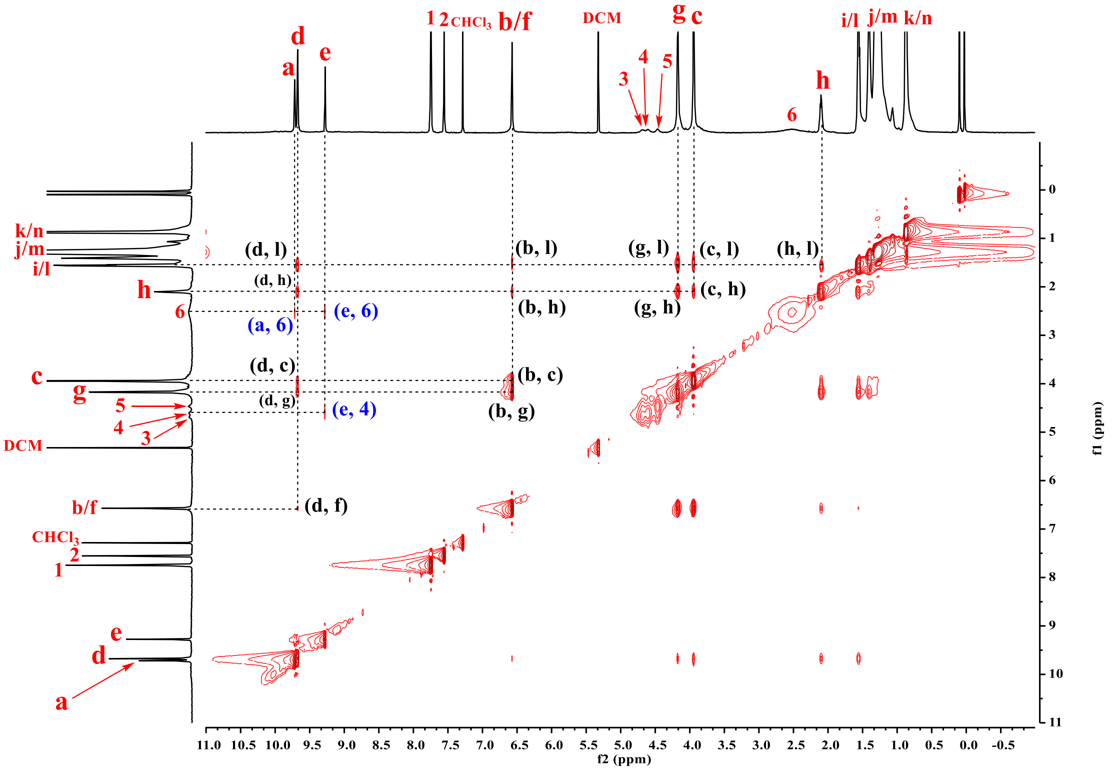

Figure S16. 2D NOESY spectrum of a mixture of 10 mM **1a** and 10 mM **L1** (600 MHz, $CDCl_3$, 298 K, mixing time = 0.4 s).

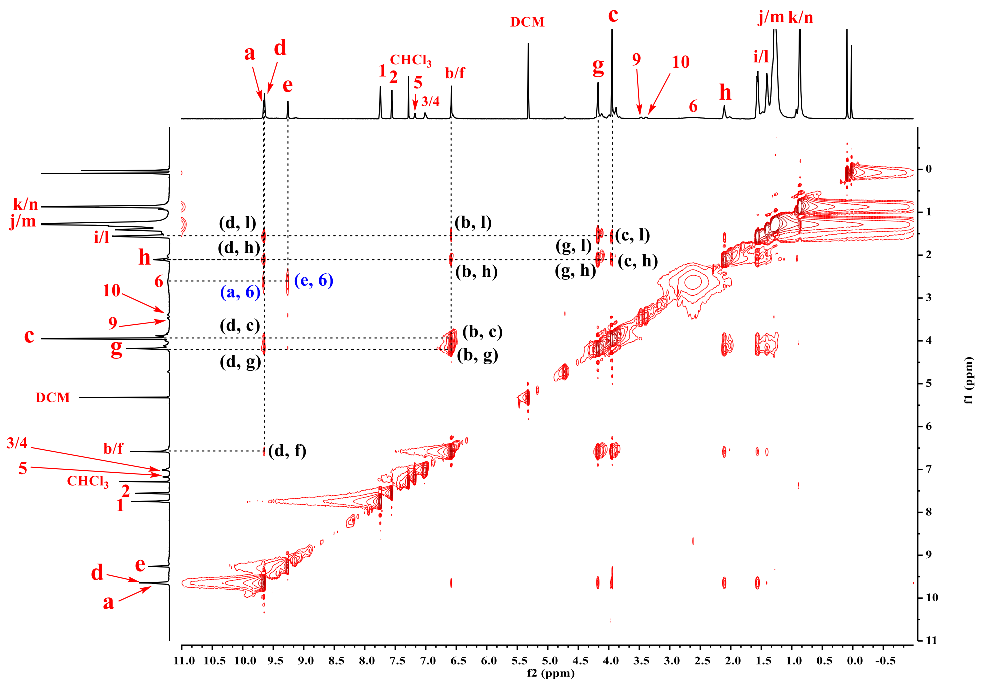

Figure S17. 2D NOESY spectrum of a mixture of 10 mM **1a** and 10 mM **L2** (600 MHz, $CDCl_3$, 298 K, mixing time = 0.4 s).

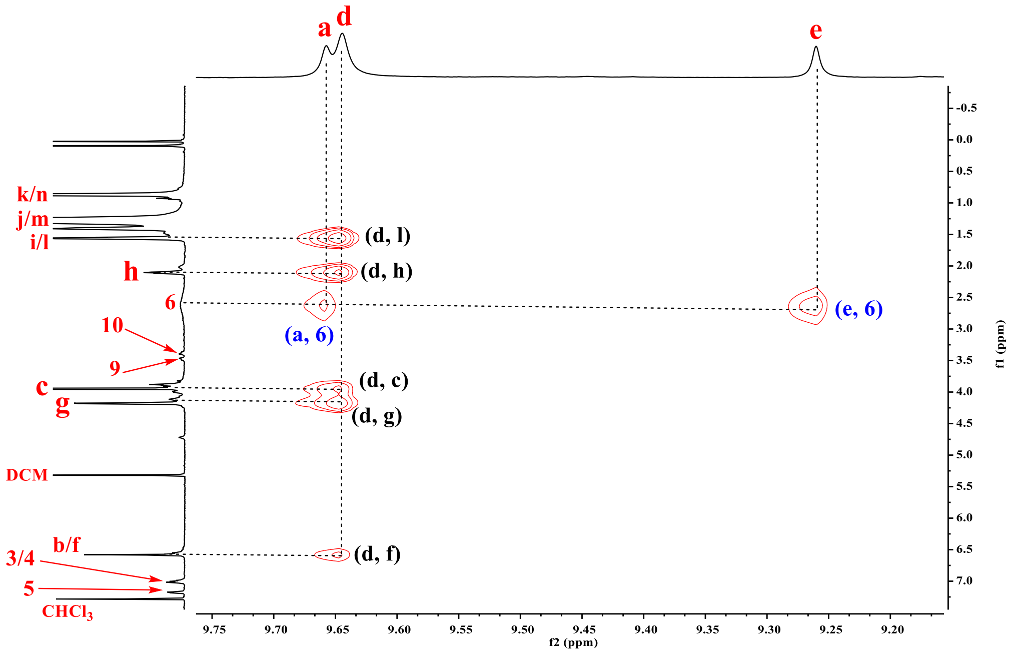

Figure S18. Partial 2D NOESY spectrum of a mixture of 10 mM **1a** and 10 mM **L2** (600 MHz, $CDCl_3$, 298 K, mixing time = 0.4 s).

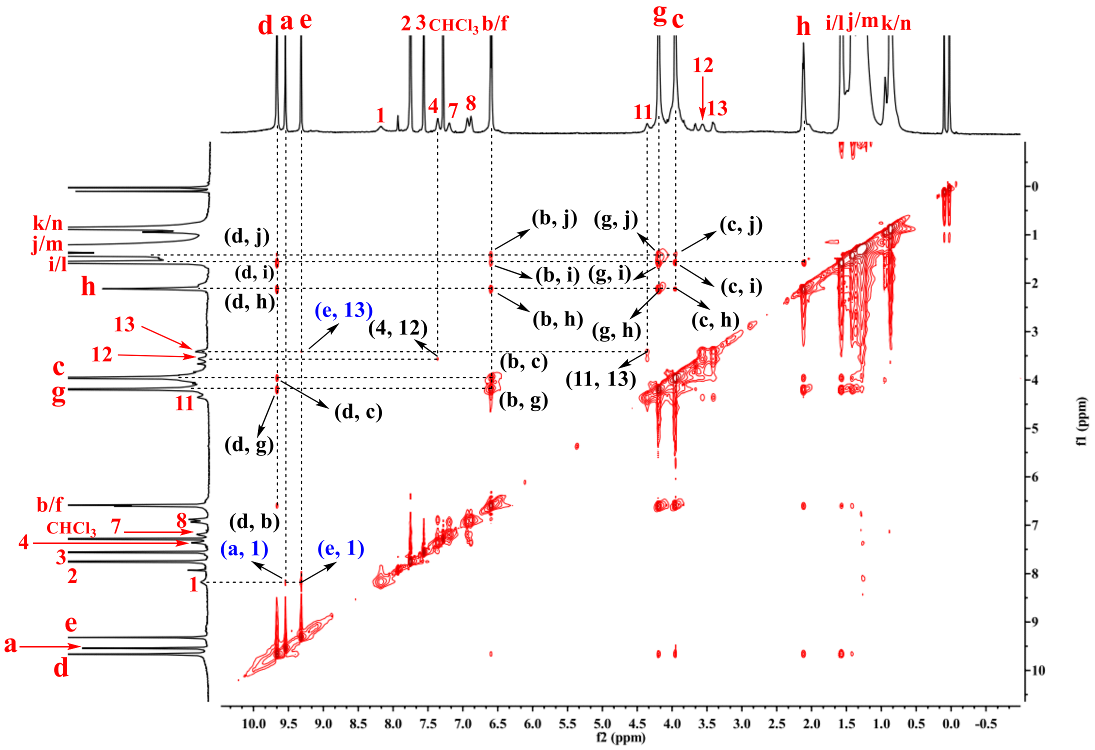

Figure S19. 2D NOESY spectrum of a mixture of 10 mM **1a** and 10 mM **L3** (600 MHz, $CDCl_3$, 298 K, mixing time = 0.4 s).

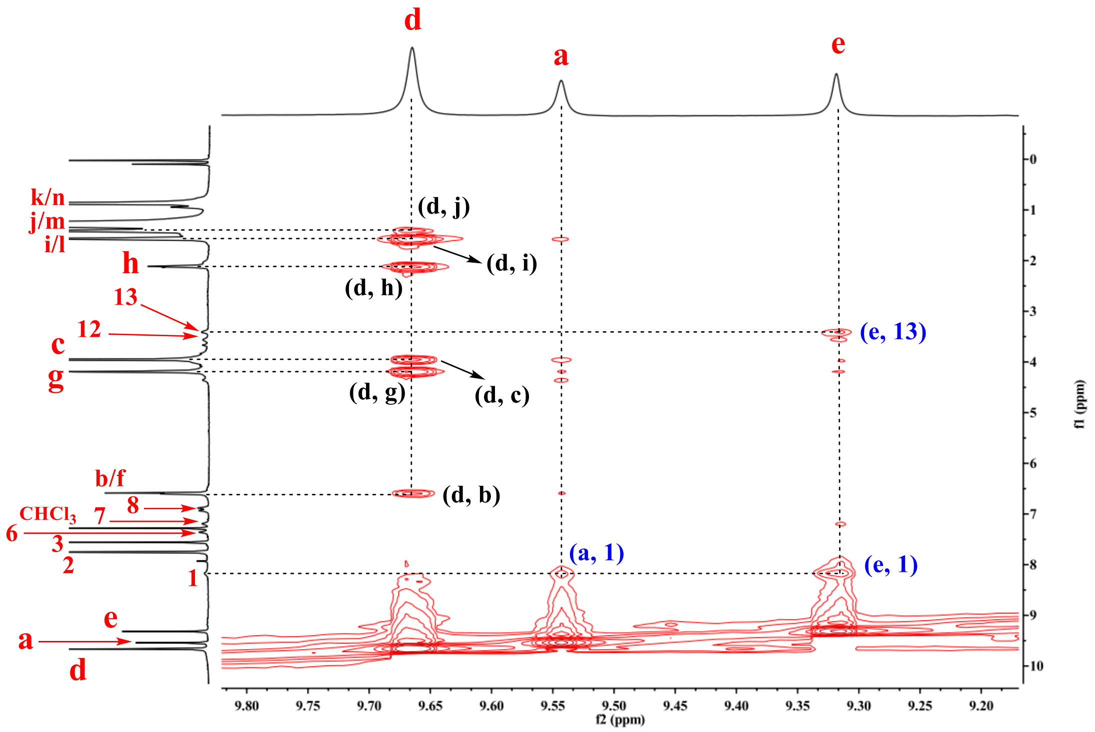

Figure S20. Partial 2D NOESY spectrum of a mixture of 10 mM **1a** and 10 mM **L3** (600 MHz, $CDCl_3$, 298 K, mixing time = 0.4 s).

8. Binding ratios determination between 1a and L1-L3

8.1 Job Plot experiments

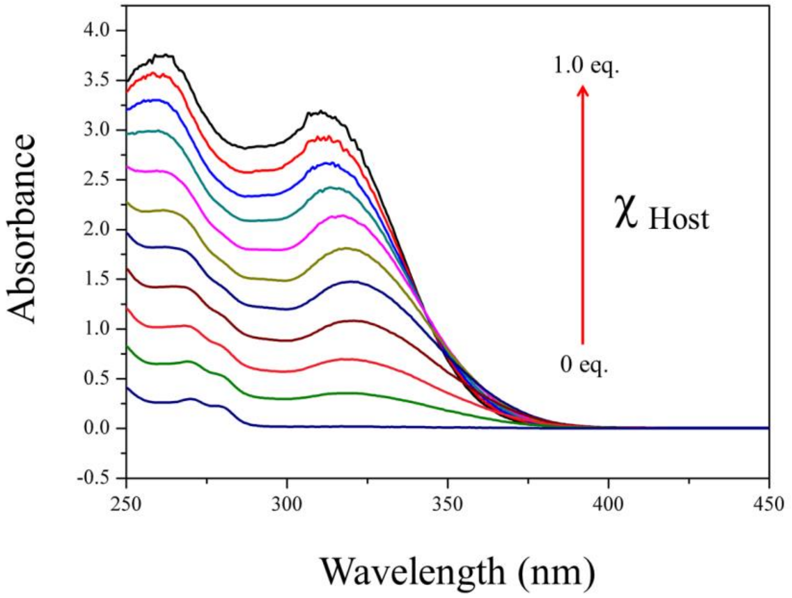

Figure S21. Stacked UV-vis spectra of Job plot experiment between **1a** and **L1** in HPLC chloroform. The total concentration is 50 μM.

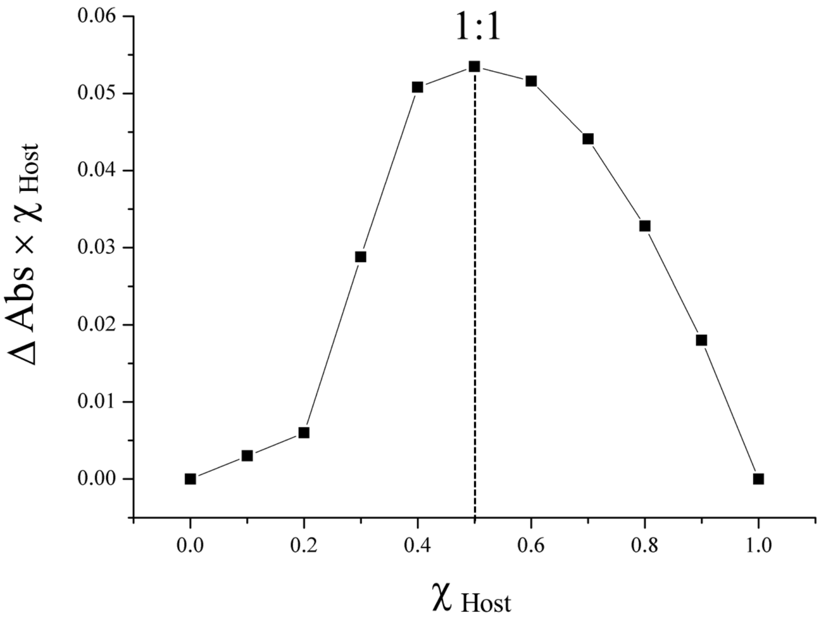

Figure S22. Job plot for the complexation of **1a** and **L1** based on the absorbance at 370 nm of UV-vis spectra.

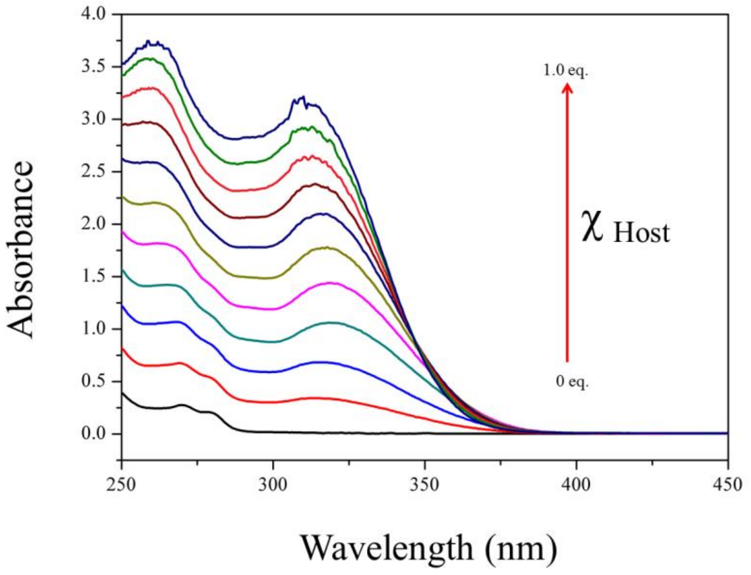

Figure S23. Stacked UV-vis spectra of Job plot experiment between **1a** and **L2** in HPLC chloroform. The total concentration is 50 μM.

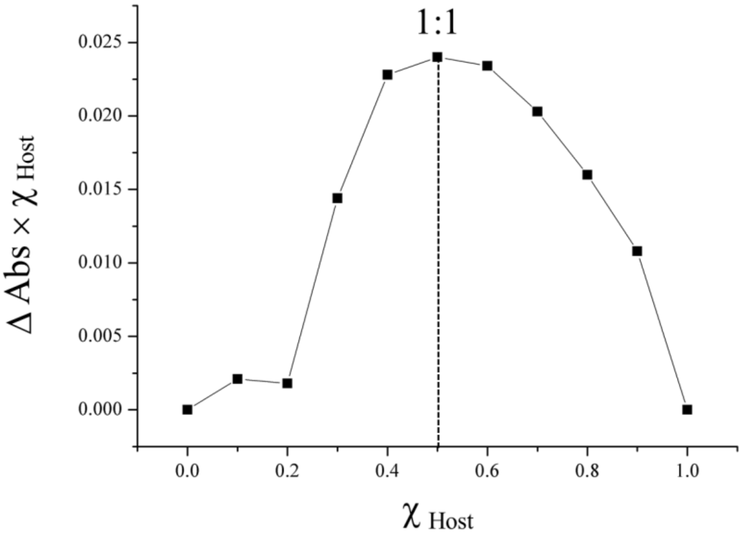

Figure S24. Job plot for the complexation of **1a** and **L2** based on the absorbance at 375 nm of UV-vis spectra.

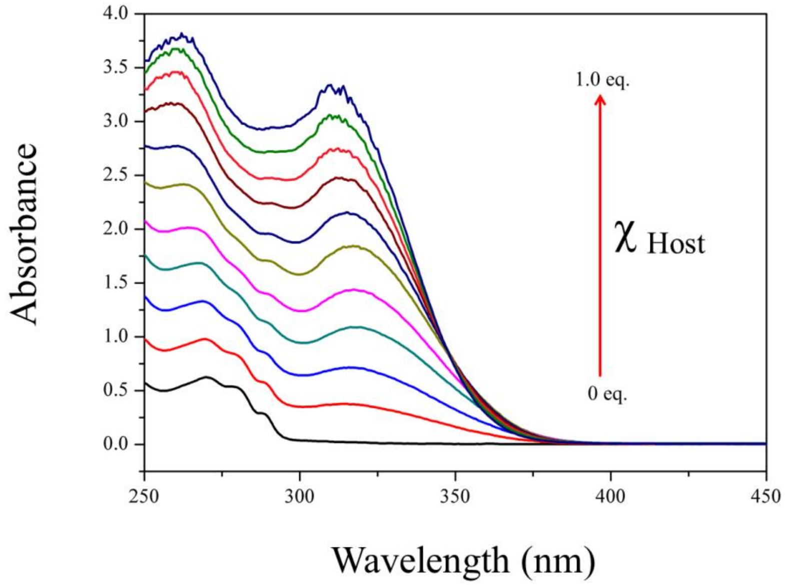

Figure S25. Stacked UV-vis spectra of Job plot experiment between **1a** and **L3** in HPLC chloroform. The total concentration is 50 μM.

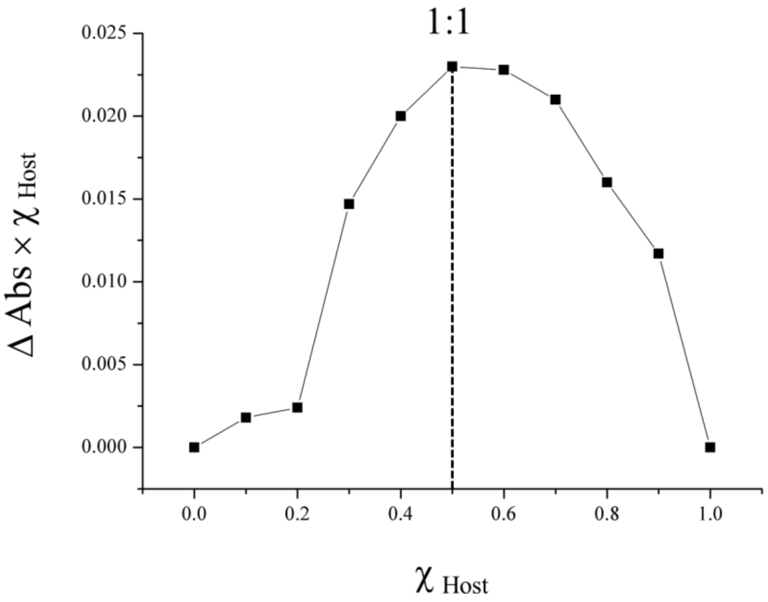

Figure S26. Job plot for the complexation of **1a** and **L3** based on the absorbance at 375 nm of UV-vis spectra.

8.2 MALDI-TOF-MS experiments

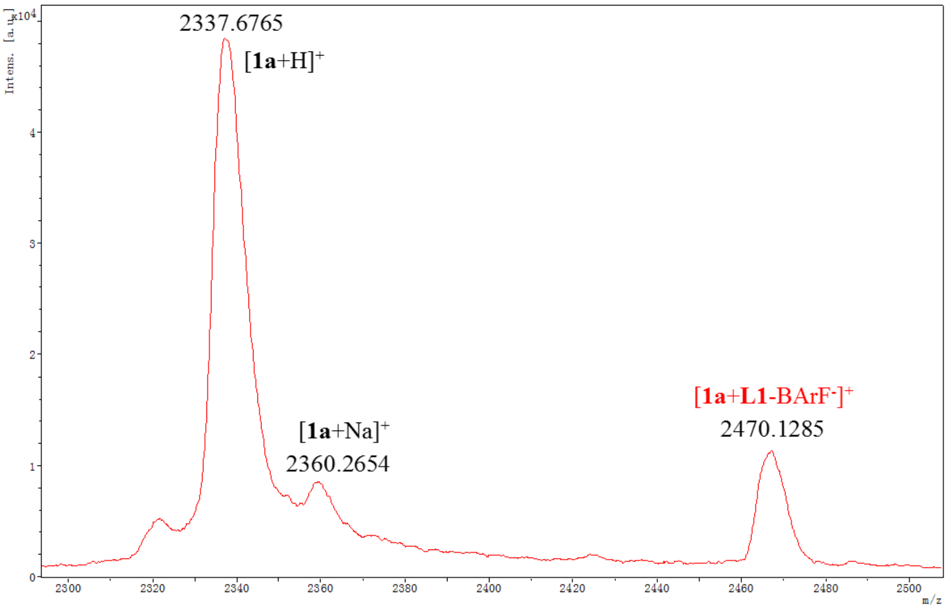

Figure S27. Partial MALDI-TOF-MS spectrum of complex **1a⊃L1**.

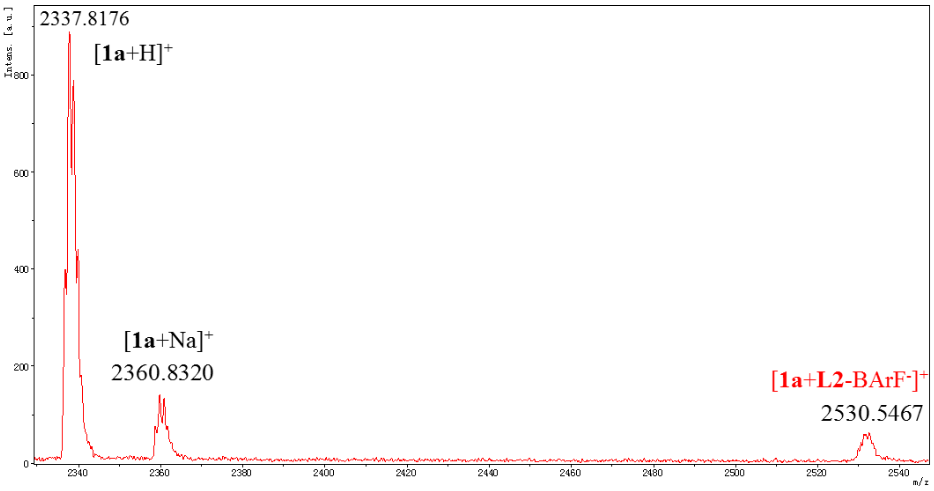

Figure S28. Partial MALDI-TOF-MS spectrum of complex **1a⊃L2**.

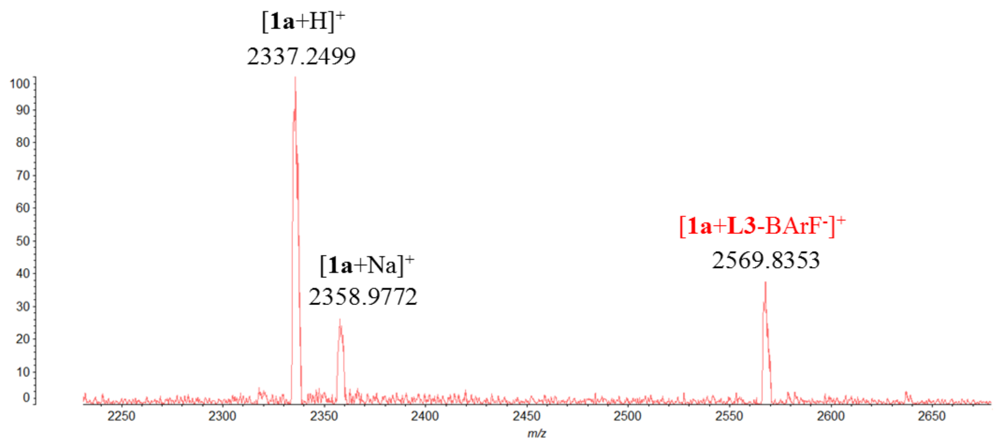

Figure S29. Partial MALDI-TOF-MS spectrum of complex **1a⊃L3**.

9. Binding constants determination between 1a and L1-L3

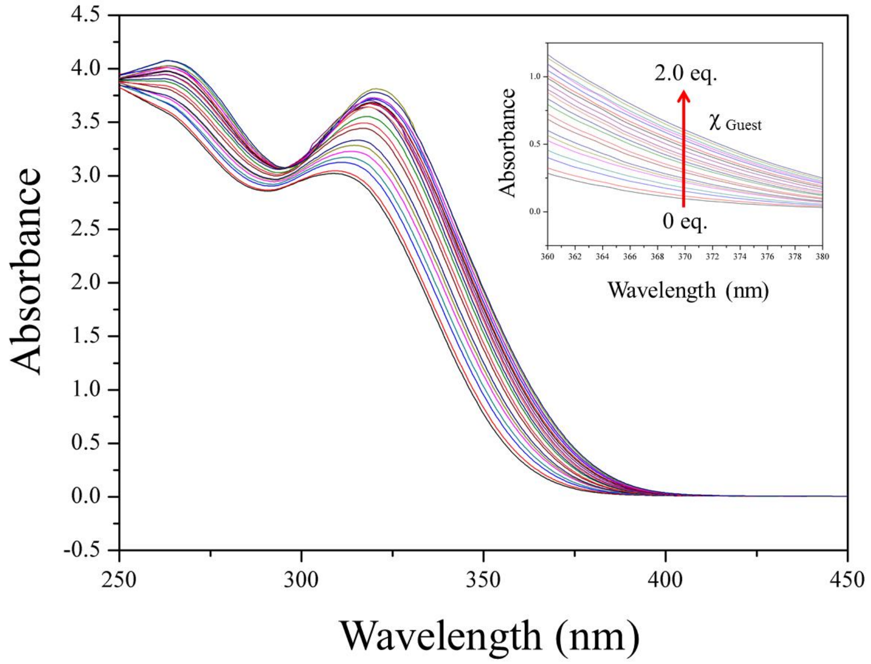

Figure S30. Stacked UV-Vis spectra of macrocycle **1a** (50 μM) titrated by **L1** from 0 eq. to 2.0 eq. in chloroform at 298 K.

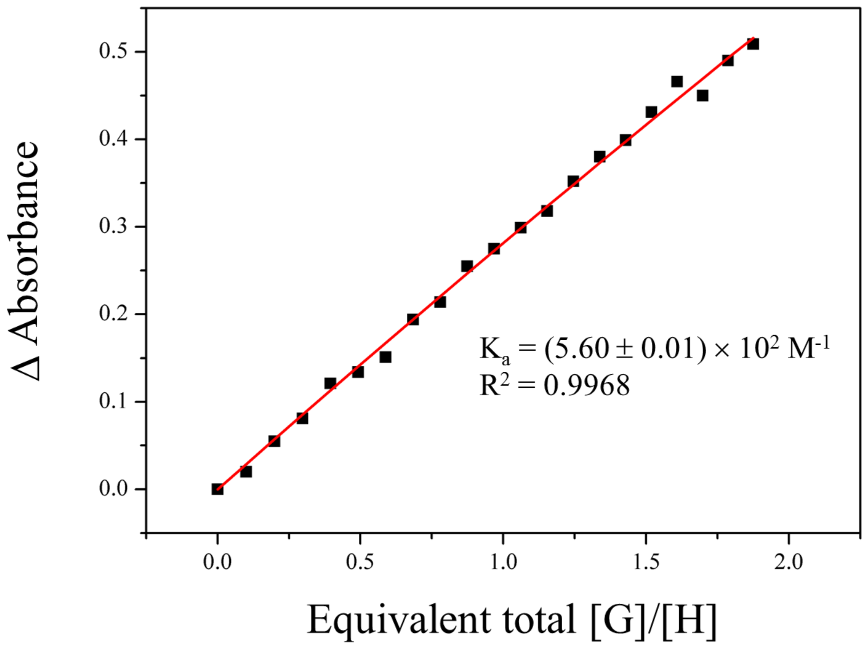

Figure S31. Determination of the binding constant of **1a⊃L1** in chloroform at 298 K. Fitting result based on absorbance at 370 nm.

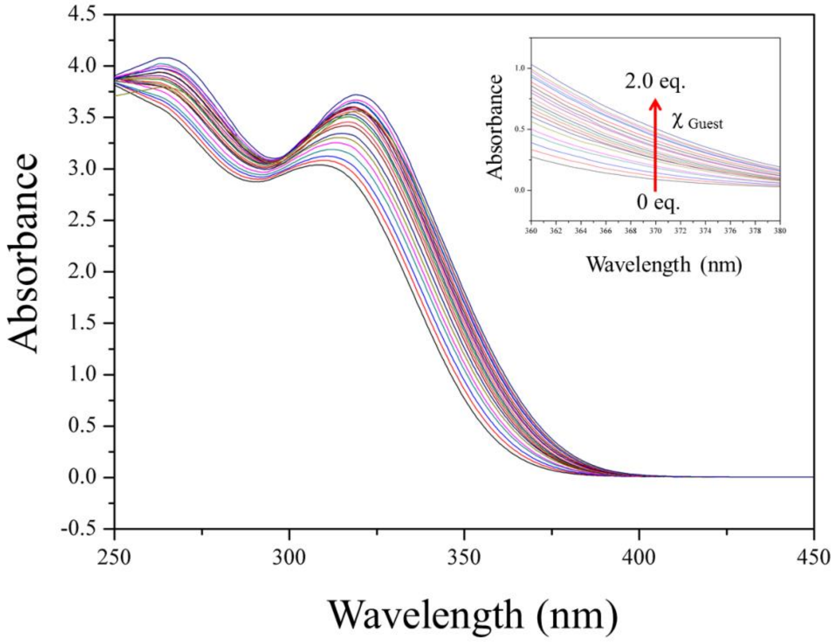

Figure S32. Stacked UV-Vis spectra of macrocycle **1a** (50 μM) titrated by **L2** from 0 eq. to 2.0 eq. in chloroform at 298 K.

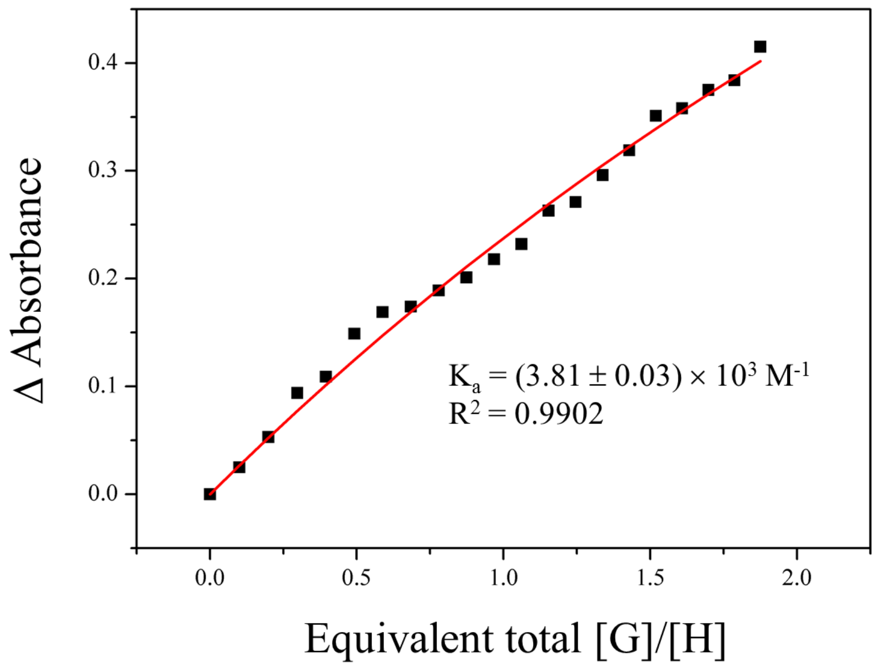

Figure S33. Determination of the binding constant of **1a⊃L2** in chloroform at 298 K. Fitting result based on absorbance at 370 nm.

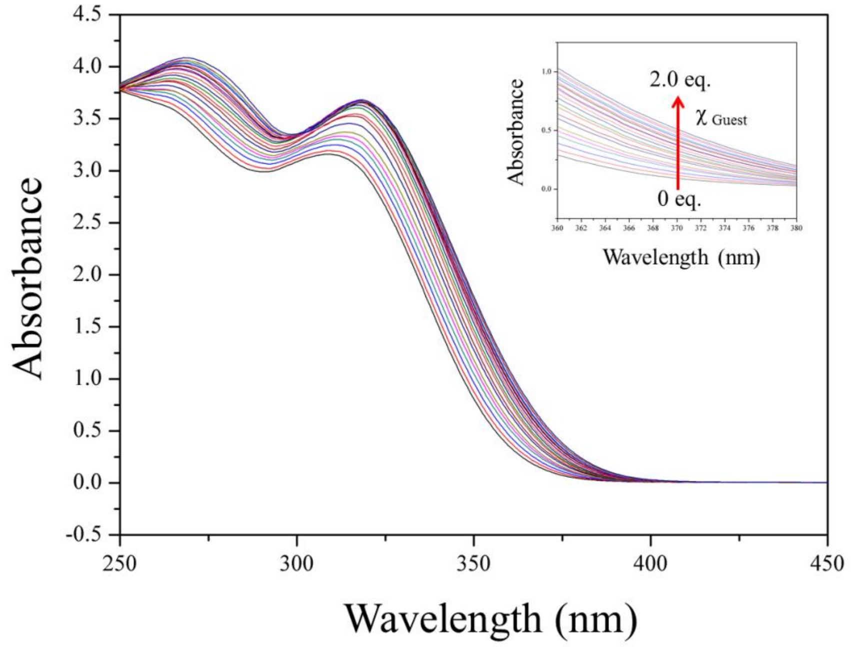

Figure S34. Stacked UV-Vis spectra of macrocycle **1a** (50 μM) titrated by **L3** from 0 eq. to 2.0 eq. in chloroform at 298 K.

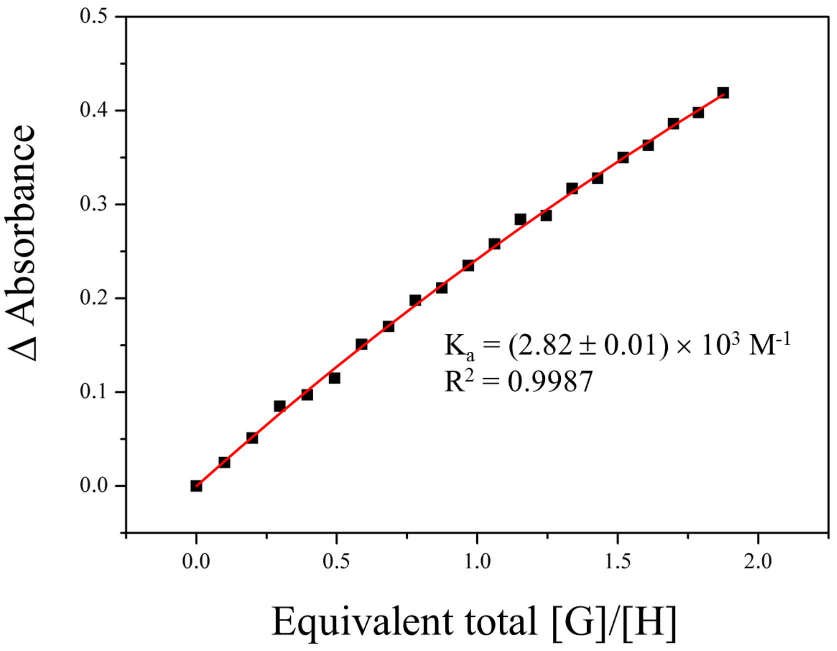

Figure S35. Determination of the binding constant of **1a⊃L3** in chloroform at 298 K. Fitting result based on absorbance at 370 nm.

10. Symmetrical CD spectral profiles of 1a, 1a⊃L/D-amino acid esters and L/D-amino acid esters

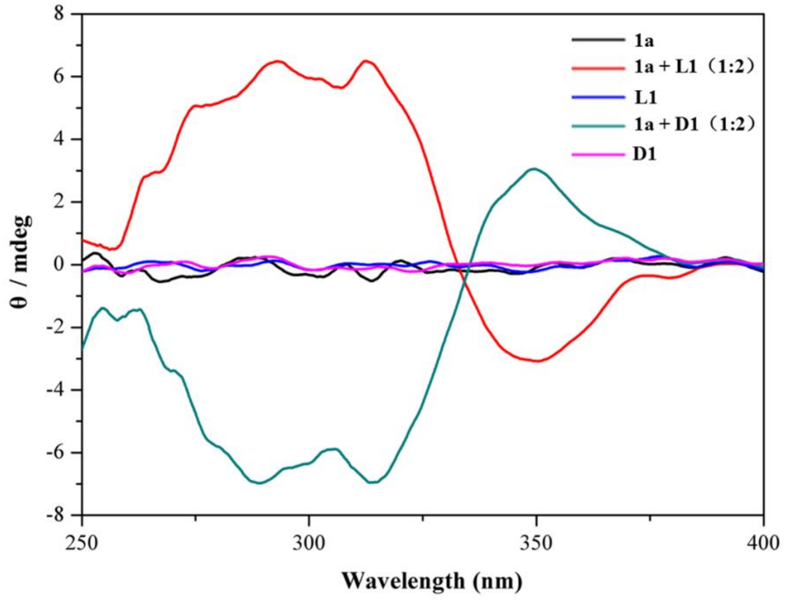

Figure S36. CD spectra of **1a** (25 μM), **L1** (25 μM), **D1** (25 μM) and **1a** in the presence of **L1** and **D1** respectively in $CHCl_3$ at 298 K (The stoichiometry of host and guest is 1:2. Optical path of cell: 10 mm).

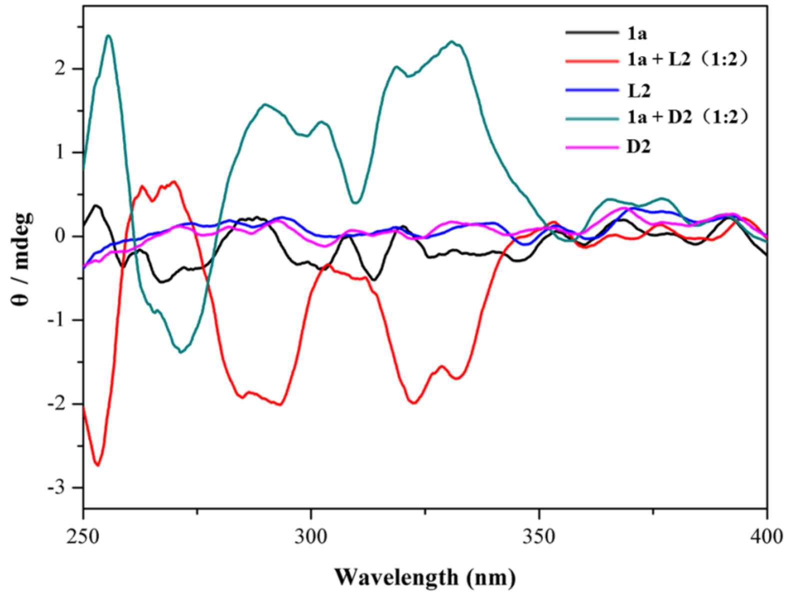

Figure S37. CD spectra of **1a** (25 μM), **L2** (25 μM), **D2** (25 μM) and **1a** in the presence of **L2** and **D2** respectively in $CHCl_3$ at 298 K (The stoichiometry of host and guest is 1:2. Optical path of cell: 10 mm).

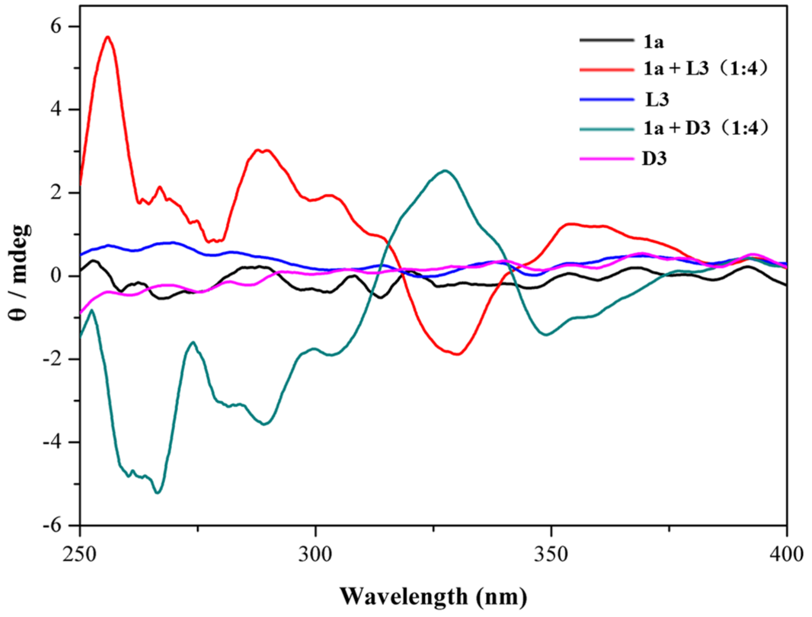

Figure S38. CD spectra of **1a** (25 μM), **L3** (25 μM), **D3** (25 μM) and **1a** in the presence of **L3** and **D3** respectively in $CHCl_3$ at 298 K (The stoichiometry of host and guest is 1:4. Optical path of cell: 10 mm).

11. DFT simulations

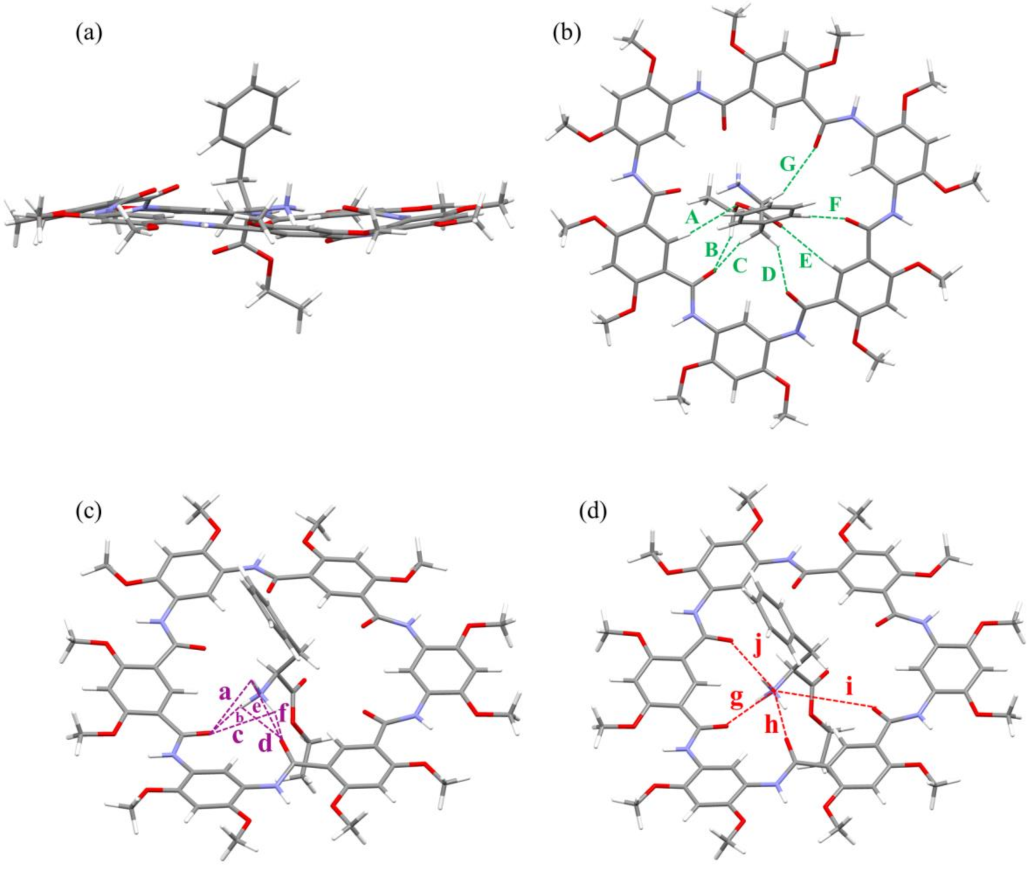

Figure S39. Side view (a) and top views (b) / (c) / (d) of optimized geometry of complex **1b$\supset$L2** at the B3LYP/6-311G (d, p) level (gray = C, white = H, red = O and blue = N). All side chains are replaced by methyl groups for simplicity and the counterion tetrakis [3,5-bis(trifluoromethyl)phenyl] borate anion ($BArF^-$) is omitted for clarity. The dashed green lines indicate C-H···O hydrogen bonds A-G and with A = 2.949 Å (150.84°), B= 2.587 Å (131.47°), C = 2.963 Å (135.27°), D = 2.411 Å (144.15°), E = 3.277 Å (122.50°), F = 2.333 Å (173.98°), G = 2.652 Å (154.95°). The dashed purple lines indicate N-H···O hydrogen bonds a-f and with a = 3.056 Å (64.01°), b = 1.814 Å (150.85°), c = 3.026 Å (65.65°), d = 3.060 Å (64.79°), e = 3.053 Å (65.11°), f = 1.827 Å (151.47°). The dashed red lines indicate N^+···O cation-dipole interactions g-j and with g = 4.033 Å, h = 2.765 Å, i = 2.781 Å and j = 4.628 Å.

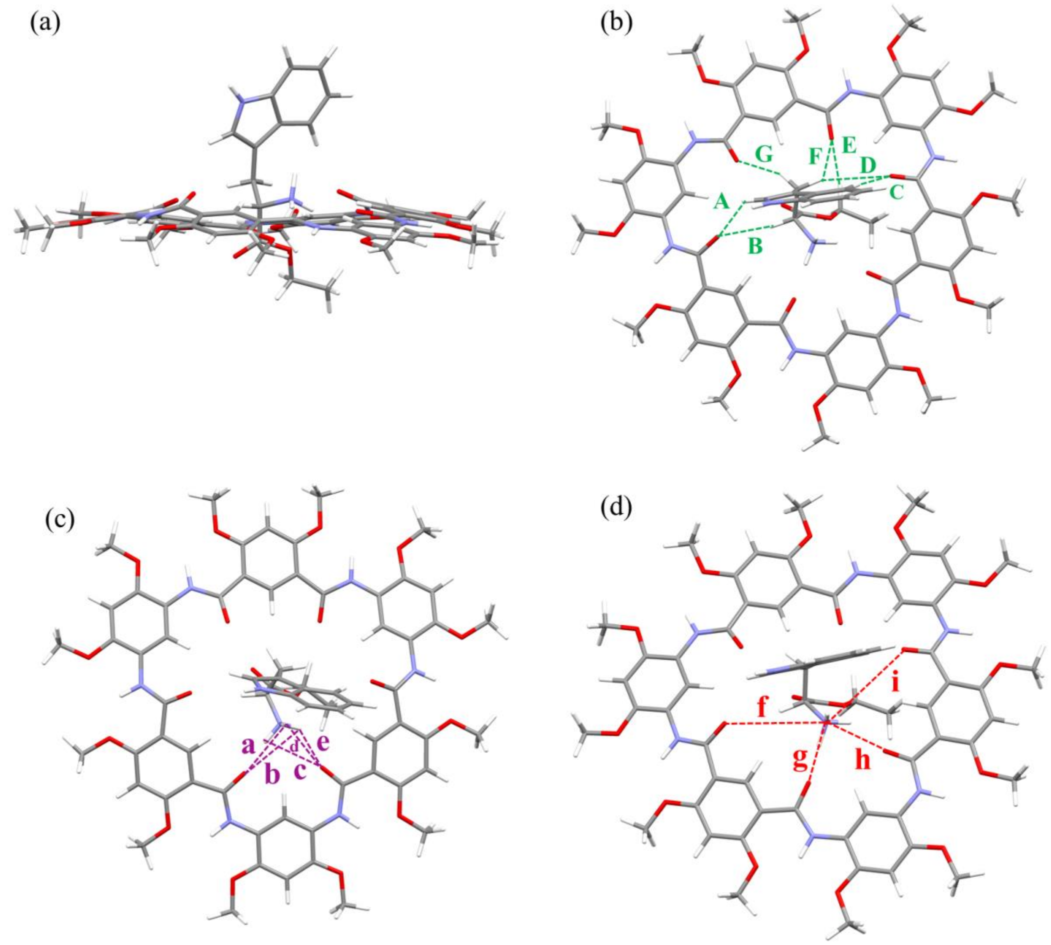

Figure S40. Side view (a) and top views (b) / (c) / (d) of optimized geometry of complex **1b⊃L3** at the B3LYP/6-311G (d, p) level (gray = C, white = H, red = O and blue = N). All side chains are replaced by methyl groups for simplicity and the counterion tetrakis [3,5-bis(trifluoromethyl)phenyl] borate anion ($BArF^-$) is omitted for clarity. The dashed green lines indicate C-H···O hydrogen bonds A-G and with A = 2.723 Å (136.37°), B= 2.521 Å (169.89°), C = 2.541 Å (173.77°), D = 3.221 Å (159.00°), E= 2.505 Å (145.68°), F = 3.104 Å (120.84°), G = 2.254 Å (155.43°). The dashed purple lines indicate N-H···O hydrogen bonds a-e and with a = 3.077 Å (61.53°), b = 3.208 Å (54.44°), c = 2.833 Å (77.10°), d = 3.013 Å (67.71°), e = 2.038 Å (127.88°). The dashed red lines indicate N^+···O cation-dipole interactions f-i and with f = 4.349 Å, g = 2.740 Å, h = 2.791 Å and i = 4.556 Å.
